# A small-dataset-trained deep learning framework for identifying atoms on transmission electron microscopy images

**DOI:** 10.1038/s41598-023-29606-9

**Published:** 2023-02-14

**Authors:** Yuan Chen, Shangpeng Liu, Peiran Tong, Ying Huang, He Tian, Fang Lin

**Affiliations:** 1 College of Electronic Engineering, South China Agricultural University, Guangzhou, 510642 Guangdong China; 2 State Key Laboratory of Silicon Materials, School of Materials Science and Engineering, Zhejiang University, Hangzhou, 310027 Zhejiang China

**Keywords:** Characterization and analytical techniques, Nanoscale materials

## Abstract

To accurately identify atoms on noisy transmission electron microscope images, a deep learning (DL) approach is employed to estimate the map of probabilities at each pixel for being an atom with element discernment. Thanks to a delicately-designed loss function and the ability to extract features, the proposed DL networks can be trained by a small dataset created from approximately 30 experimental images, each with a size of 256 × 256 pixels^2^. The accuracy and robustness of the network were verified by resolving the structural defects of graphene and polar structures in PbTiO_3_/SrTiO_3_ multilayers from both the general TEM images and their imitated images on which intensities of some pixels lost randomly. Such a network has the potential to identify atoms from very few images of beam-sensitive material and explosive images recorded in a dynamical atomic process. The idea of using a small-dataset-trained DL framework to resolve a specific problem may prove instructive for practical DL applications in various fields.

## Introduction

Identifying the atomic positions in the plane of transmission electron microscope (TEM) images at atomic resolution with the precision of a picometer or sub-picometer is a key issue to the solution of characterizing the properties of a nanomaterial. Such a task is beneficial to material research including crystal structure characterization^1–3^, atomic polarization of polar structures^4–6^, stress and strain^7^, defect or surface mitigation^3,8^, and sequential catalyst reactions^9,10^, etc. However, the conventional methods for identifying the positions of atoms^11–15^ heavily depend on the quality of the acquired images and the factors affecting image quality include the inevitable noise, the intensities relating to the ability of atoms to scatter electrons in the scanning transmission electron microscope (STEM) images, and the lens distortion in the high-resolution transmission electron microscope (HRTEM) images. What’s more, the electron dose is one of the other factors since it is limited when acquiring HRTEM images for a beam-sensitive material^16,17^.

Therefore, before using the conventional methods, a map of probabilities at each pixel for an atom that is most likely to appear (a probability map, for short) needs to be estimated first. Image-to-image mapping is one of the most adept domains of deep learning^18–24^. To our best knowledge, the existing deep-learning-driven studies for generating the probability map using the Fully Convolutional Networks (FCNs) are all based on the U-Net framework associated with a mean square error (MSE) loss function^21–24^. To train these networks, a training set must contain a lot of input–output pairs, in which the term “input” refers to one TEM image and the term “output” is its probability map which denotes the probabilities for each pixel being an atom. However, a typical number of approximately 2000 input–output pairs^21,22^ or pairs generated from 500 random atomic structures^23^ are required to construct a dataset. As a result, it is more feasible to train on a simulated image dataset. Furthermore, to better predict the experimental images with higher accuracy, iterative retraining is required. Thus, an updated training set should include the input–output pairs created from some experimental images, which have been able to be predicted well from the previous trained model. In the mentioned methods, making datasets containing a such number of input–output pairs may be one disadvantage. This is because the experimental images and their outputs cannot always be obtained easily, especially when the experimental images are contaminated by heavy noise or the number of TEM images of the beam-sensitive specimen is very limited.

In this paper, a new deep learning algorithm based on the generative adversarial networks (GANs)^25^, called atom-predicting generative adversarial networks (AP-GANs), is proposed to predict the probability map of TEM images, which has a hybrid loss function and can be trained by a small training set. In applications, the performance of AP-GANs is tested by the images and their imitated images on which intensities of some pixels were lost randomly.

## Methods

**Figure 1:**
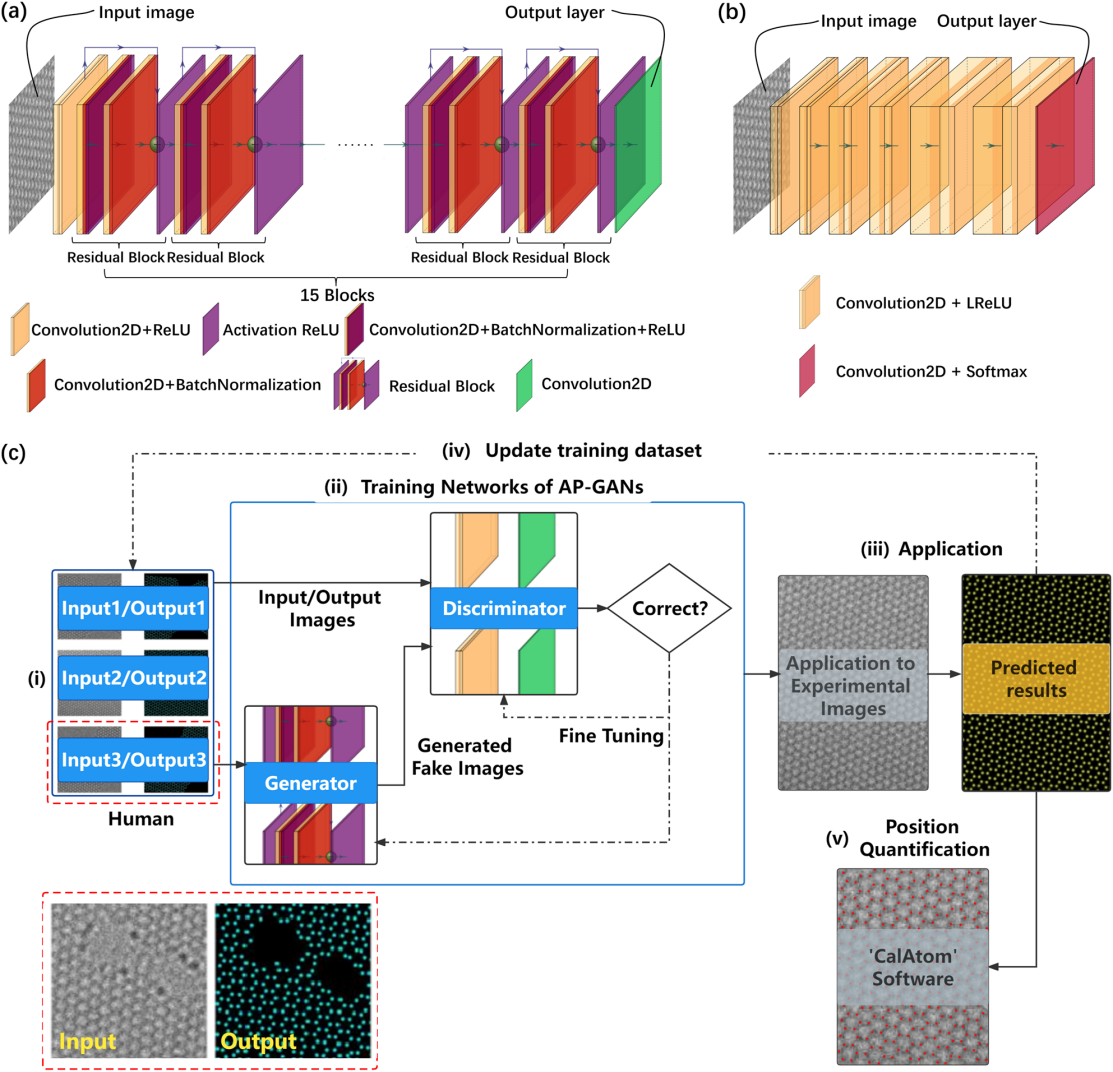
Application of AP-GANs towards identifying atomic columns on images at the atomic scale. (**a**, **b**) Schematic architectures of (**a**) generator and (**b**) discriminator in AP-GANs and disassemble layers are at the below. The green ball represents the input and the output of a residual block are added element by element. In (**b**), a thicker yellow layer represents more filters involved in Convolution2D. (**c**) Schematics of the “semi-supervised” approach. One schematic input–output pair in the training set is enclosed by a red dashed box in the bottom left corner.

The AP-GANs consist of two sub-models: a generator model and a discriminator model. As shown in Fig. 1a, the generator model generates fake images resembling the output images, whose principle is to estimate the probability map. The discriminator model in Fig. 1b is to distinguish fake images from the output images. Due to their different characteristics, the generator model and the discriminator model are often jointly trained, but each model has its own architecture and loss functions.

The training set can be composed of simulated input–output image pairs only, experimental input–output image pairs only, or a mix of them. The input image is the simulated or experimental TEM image, and the output image is the probability map on which solid points are plotted on the positions of each atom and the background is black. One schematic input–output image pair is illustrated in the bottom left box in Fig. 1c. To prepare the output image of an experimental image, the position of atoms should be identified by using the traditional methods^11–15^ or with the assistance of CalAtom software^26^, and then solid points are drawn according to these positions. If the experimental images are too noisy to identify the atomic positions reliably, atomic positions can be identified in caution from the filtered images^27,28^, while the input images are the raw experimental images. Alternatively, the training set can include only the simulated image pairs and the simulation conditions of the input images should be as similar as possible to the experimental images. When preparing a simulated image pair, the output image is drawn according to its atomic structure on the projection plane and the input image is simulated from this atomic structure. In this work, images are simulated by using ToTEM software^29^.

The architecture of the generator model mainly consists of 15 residual blocks, which contain the convolutional layers 'Convolution2D' for feature extraction and the batch normalization layers for speeding up the training process and preventing overfitting, as shown in Fig. 1a. Each residual block applies a technique called skip connections to alleviate the vanishing/exploding gradient problems^30^. The activation function of the generator model includes the ReLU function^31^. The discriminator model simply contains the convolutional layers to extract features, whose activation functions are the Leaky ReLU function and Softmax function^31^.

To minimize the loss functions, the generator model and the discriminator model are trained until reaching a Nash equilibrium. Training network heavily depends on minimizing the loss function. In general, networks trained with a hybrid loss function often yield better prediction results than those trained with a standalone loss function^32^. To highlight the atomic columns and depress intrinsic noise in experimental images, the loss functions of the generator model are deliberate and composed of three parts with different weights (see Supplementary Eqs. (1–7) and Supplementary Table S1 for hyperparameters of network training), which are the adversarial loss of the generator model and the discriminator model, the loss of the structural similarity (SSIM)^32^, and the loss of the peak signal-to-noise ratio (PSNR)^32^. The adversarial loss is an inherent loss to compel the generator model for outputting high-quality fake images to deceive the discriminator model. The loss of SSIM is introduced to improve the performance of the generator model to identify atomic columns on the boundaries of the experimental images and promote training efficiency. The loss of the PSNR is employed to reduce the impact of noise and output fake images with good PSNR. The loss function of the discriminator model is the MSE of the output images and fake images.

The flowchart of a semi-supervised training approach based on AP-GANs is shown in Fig. 1c. (i) Prepare the input–output image pairs for the training set. (ii) Train the network and minimize the loss functions. (iii) Feed the test set into the generator model to get the predictions. The generator model trained with a small training set such as ten or tens of pairs is used to predict the probability map of images in the test set, whose qualities depend on the complexity of the material sample. (iv) Update the training set and semi-supervision refers to this step. In the beginning, approximately ten input–output image pairs in the training set are sufficient to train the proposed AP-GANs for resolving atomic columns of one crystalline structure, such as graphene containing boundaries and defects. In order to obtain good predictions for every image in the test set, especially when the test set includes a certain number of images with specific features which are not included in the training set, pairs containing these specific features should be included to enrich the training set and to further optimize the AP-GANs. (v) Quantify the atom positions from the predictions. In this step, the conventional methods^11–15,26^ and the atom recognition program (ARP) can be used to measure the atoms on the predicted probability map (Flowchart in Supplementary Fig. S1). In the application, the trained AP-GANs can be directly applied to predict the probability map for the images in the test set. In the following sections, the training set and the test set are completely separated.

## Results

### Application on HRTEM images of graphene

A free-standing graphene is mounted onto a SiN (silicon nitride) TEM grid and heated to 800 °C to clean the amorphous contamination in a DENSsolutions heating holder in in-situ TEM, which is a FEI™ Titan G2 80–300 microscope equipped with a Cs corrector and a monochromator operating at 80 kV. Each frame of the HRTEM images is recorded by a Gatan CCD (Ultrascan 1000) with an exposure time of 1 s and a sampling rate of 0.23 Å/pixel. A total number of 30 frames of images with line defect evolution of graphene at atomic resolution are recorded. According to the measured aberrations^33^, the simulated images could be similar to the experimental images. Additionally, due to noise with a signal-to-noise-ratio (SNR) of 2.35, the carbon atoms cannot be successfully identified on raw HRTEM images, and the simulated images were contaminated by noise at the same level.

**Figure 2:**
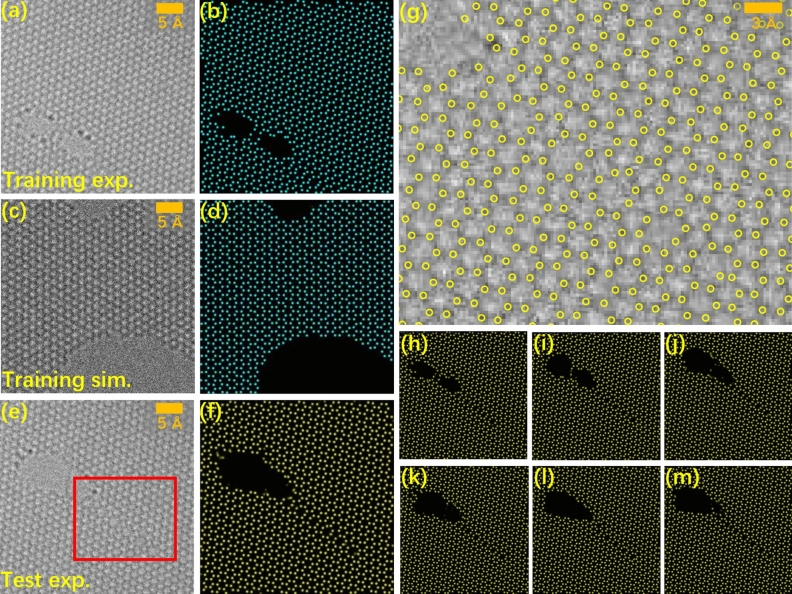
Predictions and their accuracy of modes trained via a mixture of simulated images and experimental images. (**a**–**d**) Two sets of the input–output image pairs. (**a**) An input image intercepted from one experimental image, and (**b**) the output image prepared from the atomic positions identified by using the traditional method. (**c**) A simulated input image and (**d**) the output image with atoms plotted according to its known structure. (**e**) An experimental image in the test set and (**f**) its prediction. (**g**) The region extracted from the red box in (**e**), in which the yellow circles are the positions of the atoms measured from the probability map. (**h**–**m**) Evolution of the line defects and the holes in graphene extracted from the (**h**) 1st, (**i**) 10th, (**j**) 15th, (**k**) 20th, (**l**) 25th and (**m**) 30th frames in this image series.

The training set is a combination of the simulated and experimental images, since the astigmatism was not corrected if all the input–output pairs are only collected from the experimental images. Figure 2a and c shows one sample of the experimental and simulated input–output image pairs, in which the identified atoms are plotted in green spots within a dark background, as shown in Fig. 2b and d. In the simulation, carbon atoms are shifted randomly in 3-dimensional space with an average magnitude of 10.0 pm on the projection plane along the incident-electron direction, and each TEM image was simulated from one random configuration of atoms. Therefore, the atoms are not located on the ideal hexagonal lattice, which helps to analyze the precision of the atomic position on the predicted probability map. Additionally, graphene containing line defects and irregular boundaries enriches features on images (See Supplementary Fig. S2 for more input–output pairs). It is worth mentioning that the size of the input–output images is relatively small (256*256 pixels^2^) with a sampling rate of 0.23 Å/pixel.

In this paper, an iterative training procedure was adopted. First, only 5 experimental images and 5 simulated images were randomly chosen as the input–output pairs in the training set. The AP-GANs were trained for 400 epochs and the best model was chosen to give the best prediction results for all the selected experimental images with representative features in the test set. Due to the reason that some regions containing specific features on the experimental images were unable to be predicted if applying the AP-GANs trained by the simulated input–output image pairs, more images with specific features were added for network training. The term “specific features” refers to some structural or image features, such as line defects, graphene edges, etc. In the next iteration, 5 additional experimental pairs and 5 random simulated pairs were added to the training set to retrain the AP-GANs. In this experiment, the model converged and achieved satisfactory prediction results for all the experimental images once there were 60 image pairs in total in the training set.

The generator model was able to successfully predict the probability map for the experimental and simulated images in the test set. The probability map of an experimental image of Fig. 2e is shown in Fig. 2f, whilst the highlighted region is shown in Fig. 2g. Additionally, continuous structural evolution was revealed clearly, as shown in Fig. 2h–m (the corresponding raw images are in Supplementary Fig. S3). In this structural evolution, atoms were lost and rearranged ceaselessly around the defects due to beam radiation.

**Figure 3:**
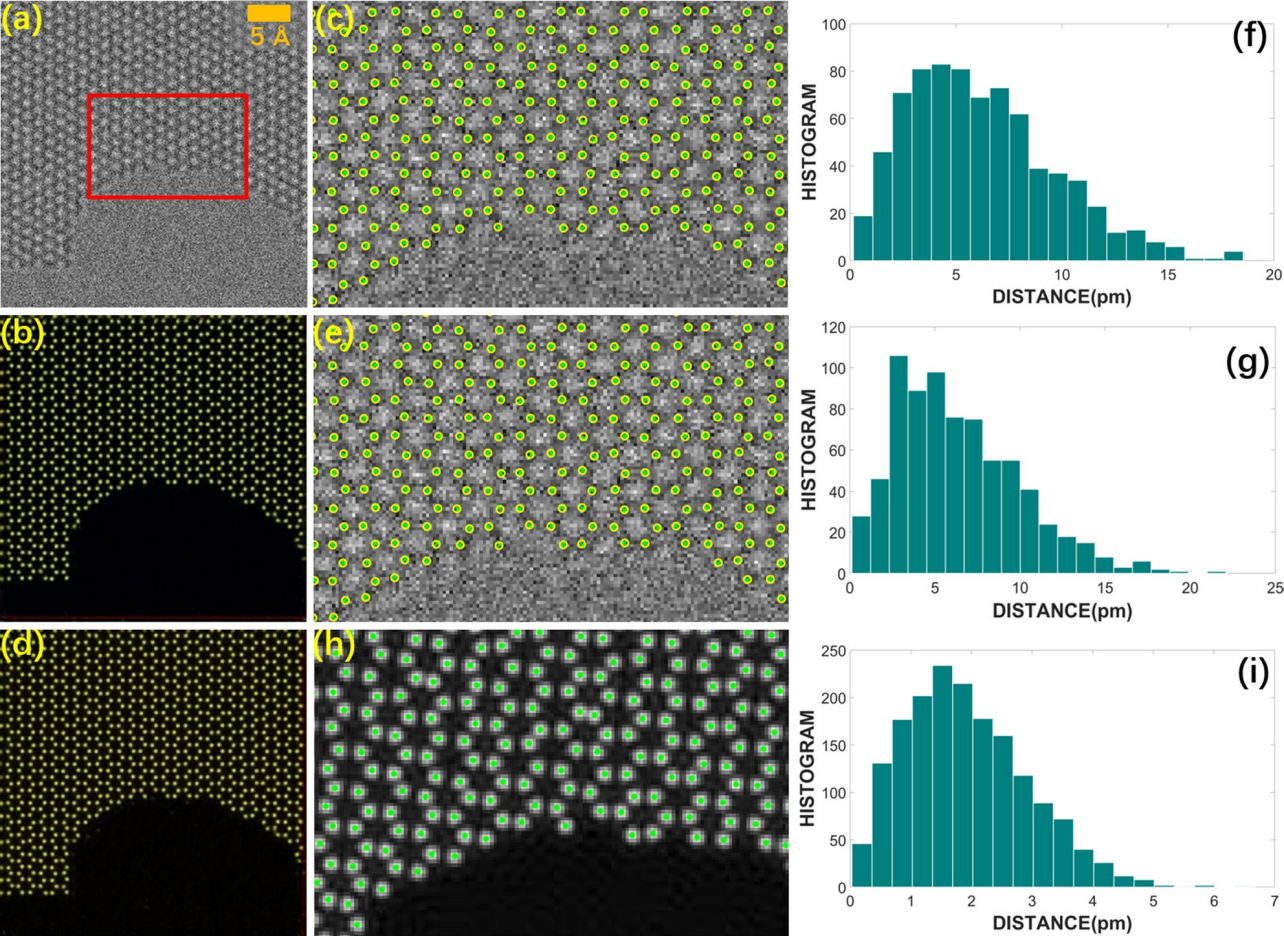
The precision of networks trained by different training sets. (**a**) A simulated image in the test set, and (**b**) its probability map predicted from the AP-GANs trained by the training set mixed with the simulated and experimental input–output image pairs. (**c**) A region extracted from (**a**), on which yellow circles are atom positions measured from (**b**). (**d**) The probability map predicted from the AP-GANs trained by the simulated training set. (**e**) A region extracted from (**a**), on which yellow circles are atom positions measured from (**d**). (**f**, **g**) Histogram of position errors between the true and those measured from the (**c**) and (**e**) image, respectively. And the errors are counted from approximately 800 positions. (**h**) Atoms are measured directly from the phase of the simulated wave of (**a**) with the assistance of CalAtom software. (**i**) Histogram of errors between the ground truth and those measured from (**h**).

Then, the precision of the atomic positions identified from the predicted probability map was measured by applying the trained AP-GANs to the simulation images in the test set. Two trained AP-GANs were tested: (i) one was trained by the training set consisting of a combination of the simulated and experimental input–output image pairs, which is the same as the AP-GANs that are used for predicting the probability map of Fig. 2e; (ii) the other AP-GANs were trained by the training set containing only 60 simulated input–output image pairs. A simulated image, its probability map predicted from the first AP-GANs and its highlighted region are shown in Fig. 3a–c, respectively. Similarly, the probability map predicted from the second AP-GANs and its highlighted region is shown in Fig. 3d and e. Atoms plotted in yellow circles were measured from the probability maps, and comparably, the ground truth positions of the atoms were known from the atomic structure and marked by the green crosses in Fig. 3c and e. For the first and second AP-GANs, the root mean square errors (RMSEs) between the measured positions of atoms and their ground truths were 6.36 ± 3.62 pm and 6.20 ± 3.43 pm, respectively. Figure 3f and g shows the histogram of position errors, in which the errors and histograms are counted from about 800 positions.

These two values are close, indicating that the training set which consists of a combination of the simulated and experimental input–output image pairs did not significantly change the precision of the probability maps predicted from the AP-GANs for the simulated images, as well as for the experimental images. It can be concluded that the precision of the probability maps predicted for the experimental images is close to these two values. And comparably, the precision is 21.54 ± 14.84 pm if applying the traditional methods to directly label atomic positions. Additionally, a statistical error, as one part of the precision, comes from the limitations of the CalAtom software and the image resolution. Figure 3h shows that the atoms measured directly from the phase of the simulated wave of Fig. 3a by using CalAtom, and Fig. 3i shows the histogram of these position errors. Measured from Fig. 3i, the bias of the atom positions is limited to 1.88 ± 1.00 pm, and it is certainly involved in the position errors when measuring the precision of the atomic position from the probability map.

The noise level, of course, affects the precision of measuring the atoms. If the AP-GANs were trained by a noisier training data set and then predicted the noisier test set, the precision of measuring atom position becomes worse. Supplementary Fig. S4 showed the simulated images of varying SNR and their predictions. For example, for the simulated image with its SNR equaling to 0.22, the precision of measuring atom position is the worst as 33.58 ± 23.89 pm; and if the image SNR equals to 1.38, the precision of the atom position measured from the predicted images is 11.94 ± 8.64 pm.

**Figure 4:**
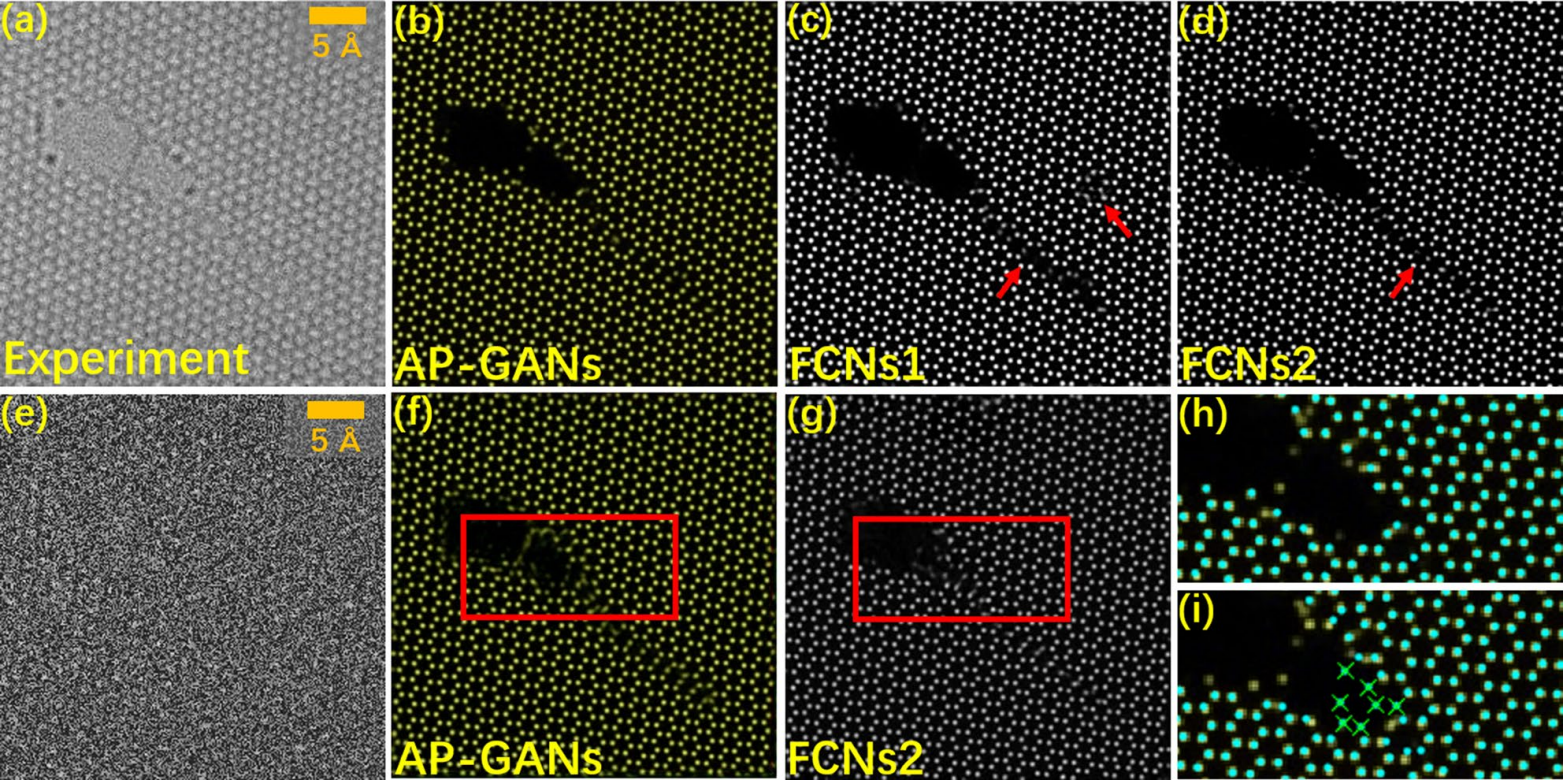
Comparison of the predictions of AP-GANs, FCNs1 and FCNs2 for the same experimental images in the test set. (**a**) The experimental image, and (**b**–**d**) their probability maps were obtained via (**b**) the proposed AP-GANs, (**c**) FCNs1 in ^21^ and (**d**) FCNs2 in ^22^. (**e**) Imitated low-dose image simulated from (**a**). (**f**, **g**) Atom maps were predicted by using (**f**) AP-GANs and (**g**) FCNs2, respectively. (**h**, **i**) Regions extracted from (**f**) and (**g**), respectively. The darker yellow dots were estimated from (**a**) normal images via using AP-GANs and FCNs2, and green dots were predicted by ARP and green crosses highlight the clear artefacts.

To further evaluate the performance of the proposed AP-GANs, a comparison is conducted to evaluate the prediction results between the AP-GANs and two FCNs-based frameworks, the results of which are shown in Fig. 4a–d. The framework used in^21^ is denoted as FCNs1, whilst another architecture adopted in^22^ is denoted as FCNs2. The two FCNs-based frameworks are trained with the same 60 input–output image pairs. In Fig. 4a–d, the results indicate that the AP-GANs significantly outperform both the FCNs frameworks in identifying the atomic columns in the experimental images with defects. For instance, some atoms at the boundaries of the defects are missing in the predictions. Such a phenomenon is intensified by the prediction of FCNs1, as indicated by the red arrows in Fig. 4c–d. More experimental results can be seen in Supplementary Fig. S5.

Furthermore, the AP-GANs outperform the FCNs frameworks in terms of fewer artefacts especially when applying to images in which some pixels lost their intensities randomly. The artefacts mean that the shape and intensity of the emerged bright spots are similar to that of an atom, but in fact the central positions of these spots should not be an atom. The 30% of the pixels in each HRTEM image were randomly set to be zero values in both of the training and test sets, which was attempted to imitate the low-dose images. Should be mentioned that, some pixels were completely dark in these imitated images, which is the common feature as the real low-dose image; but the noise statistical characteristics of the imitated images may be different from the real low-dose image. All the networks are retrained afterward. In Fig. 4e, an imitated low-dose image of the test set is illustrated, in which there exists nearly no lattice or vacancies. In fact, Fig. 4e is generated from Fig. 4a by reducing the SNR from 2.35 to 0.03, but it becomes evidently difficult to predict the atom positions. The results obtained by AP-GANs and FCNs2 are shown in Fig. 4f and g, respectively, from which we may conclude that both of the approaches have the potential for atom position predictions. However, at the boundary regions in Fig. 4h and i extracted from Fig. 4f and g, we found the atoms predicted by AP-GANs are better: fewer artefacts that are marked by green crosses, and fewer atoms are missing compared with the prediction from the normal image. The position precision of the maps obtained by AP-GANs is 11.22 ± 6.13 pm, while that of the maps obtained by FCNs2 is 13.30 ± 7.38 pm. Such precision values were counted from a total of 10 images in the test set and the artefacts marked by green crosses on FCNs2 results in Fig. 4i were excluded. Additionally, artefacts that appeared on vacuum regions predicted by FCNs2 are illustrated for both the simulated and experimental images in Supplementary Fig. S6 and Supplementary information 1 and 2.

### Atom identification with element discernment on STEM images of PbTiO_3_/SrTiO_3_ multilayers

**Figure 5:**
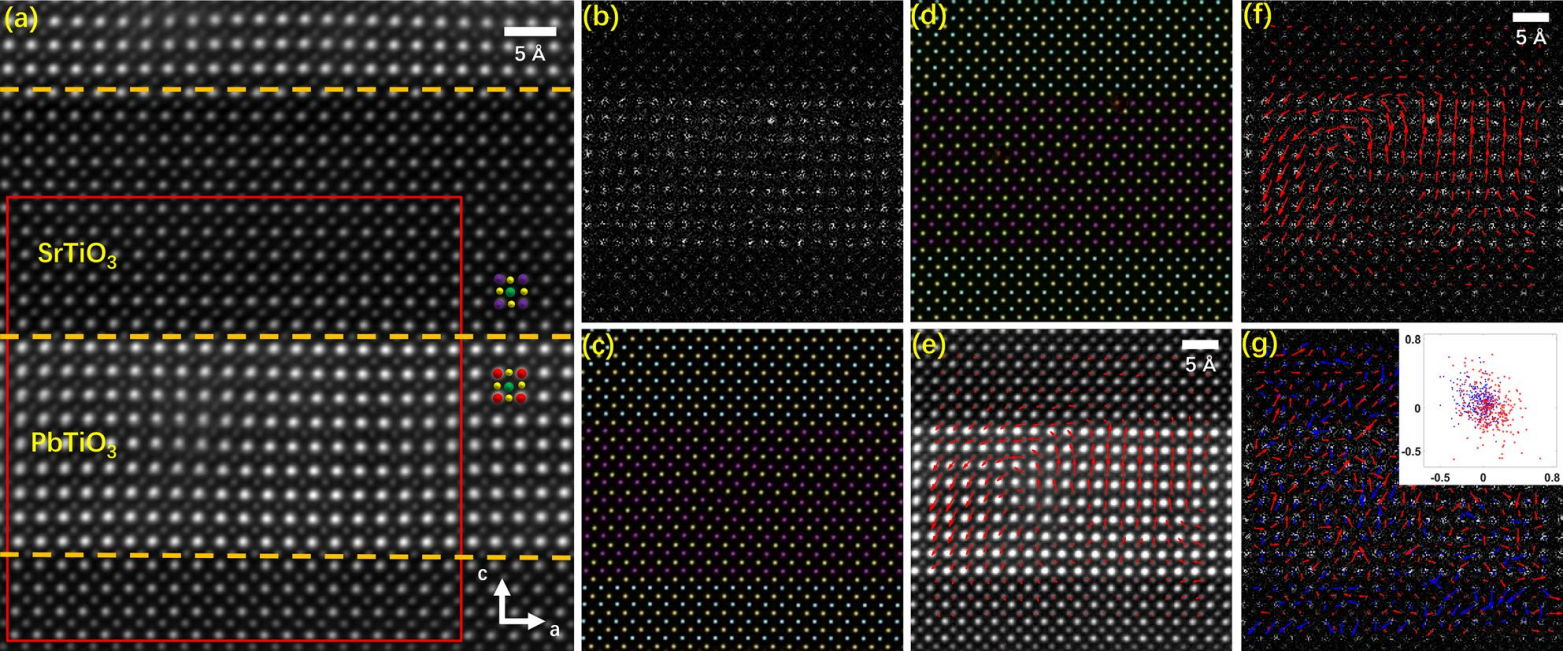
Probability map with element discernment and atom displacement vector map on STEM images of PbTiO_3_/SrTiO_3_ superlattice. (**a**) One experimental STEM image of PbTiO_3_/SrTiO_3_ superlattice in the test set. Purple, red, green, and yellow balls denote the positions of Sr^2+^, Pb^2+^, Ti^4+^, and O^2-^ columns respectively, and orange dashed lines indicate the interface between PbTiO_3_ and SrTiO_3_. (**b**) The compressed image simulated from the red region of (**a**). (**c**, **d**) Probability maps with element discernment and (**e**, **f**) Ti atom displacement vector maps relating to polarization, corresponding to the red region of (**a**) and predicted from experimental STEM images in (**c**, **e**) traditional mode and (**d**, **f**) compressed mode. (**g**) The magnified displacement of each position was obtained by comparing (**d**) with (**c**). The inserts are the absolute displacements of all positions with units of pixels. Red and blue arrows (dots) represent the Ti and Pb/Sr sites, respectively.

This experiment is conducted on STEM images of polar structures to achieve domain structure, elemental information at atomic resolution and atom displacement vector map relating to polarization. The PbTiO_3_/SrTiO_3_ multilayers, about 50 nm thick, were acquired in the high-angle annular dark field (HAADF) mode on spherical aberration corrected TEM (FEI Titan G2 80–200 ChemiSTEM) with a sampling rate of 0.29 Å/pixel and the incident electrons being along [010] direction ^4^. Figure 5a shows one of the experimental images with crystalline structures of PbTiO_3_ and SrTiO_3_ marked. This specimen was investigated via atomic-resolution EDS-mapping^4^, showing that the interface between the PbTiO3 and SrTiO3 layers is sharp at the atomic scale.

To obtain the polarization map, the positions of oxygen atoms and Ti atoms should be measured. Although the oxygen atoms cannot be detected in STEM images, the centers of Pb and Sr atoms are able to replace the centers of the Ti-'centered' oxygen atoms, such that the Pb/Sr atoms can be measured directly by CalAtom. The positions of Ti atomic columns infer the offset of the Ti-'centered' oxygen octahedra for defining the electric dipole. And the main problem is to identify the positions of Ti atoms, caused by the low intensity of Ti atoms and the disturbance from Pb/Sr atoms. Aiming at this problem, when creating the experimental pairs, the TEM images of a thin specimen were preferentially selected to prepare the input–output experimental pairs, on which the positions of Ti atoms could be identified. If some of the positions of the Ti atoms are obviously wrong due to the disturbance from the intensity of Pb/Sr atoms by using traditional methods, these positions should be corrected manually.

To train the AP-GANs, only experimental images were included in the input–output pairs, where the positions of Pb/Sr and Ti sites were quantified with the assistance of CalAtom. One input image corresponded to one output image with atoms of different species marked by different colors, and the atomic resolution EDS-mapping obtained in the experiment played an important role when we preparing the training set as it indicated the approximate range of intensity for different atomic columns. The Pb, Sr and Ti atoms were labeled by magenta, cyan and yellow color dots in the training set. As very few atoms of Pb and Sr atoms were mixed in the interface and the sample is uniformly thick, the intensity fluctuation is attributed to the elemental species of the atoms. The performance of the AP-GANs entirely depends on the characteristics of the prepared training set. In the training process, 5 input–output pairs were added to the training set at each iteration. The probability maps with element discernment predicted from the models trained by 10, 20 and 30 input–output pairs are shown in Supplementary Fig. S7, respectively.

When the number of input–output image pairs in the training process reached approximately 30, we noticed that the positions of Ti atoms on the probability map of atoms with element resolved were stabilized, comparing the predicted results with that of mutually measured by CalAtom. The probability map with element discernment corresponding to the red region of Fig. 5a is shown in Fig. 5c, which is in the test set. Furthermore, the validity of the predictions is verified by the displacement vector map of Ti atoms, which is shown in Fig. 5e and in agreement with the previous work^4^ (Also see displacement vector map of a fused region in Supplementary Fig. S8). Focusing on a small region where the positions determined from the probability map deviated with random distances from those obtained by using the traditional method and manual correction, it is interesting that the intensity of Ti atoms measured from the probability map has a better statistical performance: a slightly larger average, and a slightly narrower standard deviation. This indicates that the intensity distributions of the local Ti atoms are closer to each other, which is more reasonable. See the details about the comparison in Supplementary Fig. S9.

A similar task testing AP-GANs was carried out for the compressed images, which were attempted to imitate the images acquired in the compressive sensing (CS) mode without restoration^34^. To imitate the compressed images, we randomly chose 30% of the total image pixels to preserve their intensities while setting that of the remaining pixels to zero, which simulates a scanning along a random trajectory to reduce the STEM acquirement time. One compressed image is shown in Fig. 5b. Then, the AP-GANs were retrained. The probability maps of atoms with element discernment are shown in Fig. 5d, which is the same example as Fig. 5c but predicted from the compressed image. Despite the bad quality of the compressed image, the polarization structure agrees with that obtained on the normal image, shown in Fig. 5f. The biases between the two sets of positions are magnified and plotted as vectors for each position in Fig. 5g. The inserted figure in Fig. 5g gives the absolute biases in pixel units, showing that all of the biases are less than one pixel. Statistics show the position deviations of Ti and Pb/Sr sites are 7.0 and 5.2 pm, respectively when comparing the positions obtained from the probability maps of the compressed images with those measured from the normal-dose case. More importantly, the directions of position deviations are random in Fig. 5g. (Also see examples in Supplementary Fig. S10).

## Discussion and conclusion

We employ AP-GANs to estimate the probability map of atoms at each pixel or the probability map with element discernment, in order to identify blurred atoms especially for images in which some pixels lose their intensities randomly. The advantages of the proposed method benefit from the hybrid loss functions, the scheme of enriching the training set, and the property of AP-GANs self: (i) feature extraction from boundaries, defects, and noise suppression rely on SSIM, PSNR, and MSE in the hybrid loss functions of the generator model, while only MSE is used in the previous studies^21–23^; (ii) to enrich more features of an updated training set, the new added input–output pairs are created from the experimental images that cannot be estimated very well, which differs from the scheme used by FCNs2; (iii) the AP-GANs are able to extract more image features than FCNs, due to the deeper and more complex network architectures of AP-GANs; (iv) different from the previous deep learning frameworks, three color channels are reserved because it can be linked to elemental information on TEM image.

In order to make the AP-GANs better to predict the probability map for the experimental images, experimental input–output pairs must be included in the training set, since their features are directly related to the images to be analyzed. In this paper, only about 30 experimental images of 256*256 pixels^2^ are used in the training set, and the total pixel area of all the training set is small, only equaling to the half pixel area of one image of 2048*2048 pixels^2^. The application on graphene has proven that the AP-GANs are more reliable than the existing networks, especially when only 70% of the pixels are reserved. The probability map with element discernment and polar structure of PbTiO_3_/SrTiO_3_ multilayers are resolved with high accuracy, only reserving 30% pixels. We will be able to train a network using a smaller training set if a well-designed loss function or network is adopted, which is important for developing TEM techniques in the future. Such an application also has great impacts on some special application scenarios, e.g., single molecule imaging at the near-atomic scale, atomic structure evolution in catalytic processes relating to the energy issue, and dynamic chemical reactions in battery research, etc.

## Supplementary Information

Supplementary Information 1.

Supplementary Information 2.

Supplementary Information 3.

## Data Availability

The data of this study will be deposited on GitHub, and the codes will also be available from the corresponding authors, Professor Fang Lin (email: linfang@scau.edu.cn), upon reasonable request.
