# Supplementary figures and images for: A small-dataset-trained deep learning framework for identifying atoms on transmission electron microscopy images (part 2 of 2)
Source: Sci Rep. 2023 Feb 14;13:2631. doi: 10.1038/s41598-023-29606-9 (PMC9929221; doi:10.1038/s41598-023-29606-9)

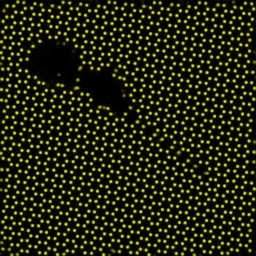

Supplement: Supplementary file 1 — Supplementary Information 1. [file 41598_2023_29606_MOESM1_ESM.zip › Attachments/alltogether_experiment/sample_hello_3dat_16_0p1_11_ganhi_single.jpg]

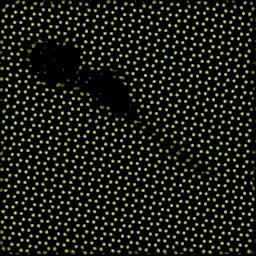

Supplement: Supplementary file 1 — Supplementary Information 1. [file 41598_2023_29606_MOESM1_ESM.zip › Attachments/alltogether_experiment/sample_hello_3dat_16_0p1_11_gan_single.jpg]

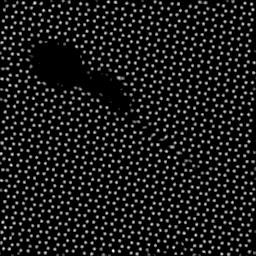

Supplement: Supplementary file 1 — Supplementary Information 1. [file 41598_2023_29606_MOESM1_ESM.zip › Attachments/alltogether_experiment/sample_hello_3dat_16_0p1_11_ifcn2_single.jpg]

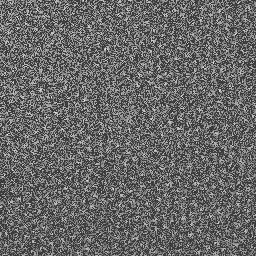

Supplement: Supplementary file 1 — Supplementary Information 1. [file 41598_2023_29606_MOESM1_ESM.zip › Attachments/alltogether_experiment/sample_hello_3dat_16_0p1_11_ori_single.jpg]

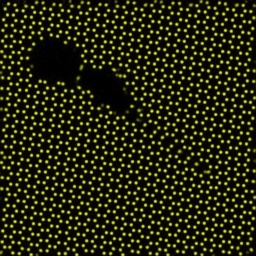

Supplement: Supplementary file 1 — Supplementary Information 1. [file 41598_2023_29606_MOESM1_ESM.zip › Attachments/alltogether_experiment/sample_hello_3dat_16_0p1_12_ganhi_single.jpg]

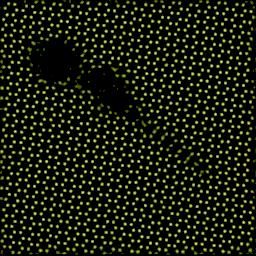

Supplement: Supplementary file 1 — Supplementary Information 1. [file 41598_2023_29606_MOESM1_ESM.zip › Attachments/alltogether_experiment/sample_hello_3dat_16_0p1_12_gan_single.jpg]

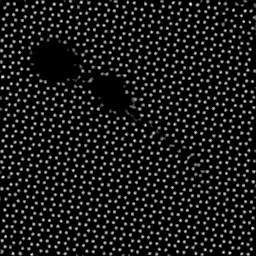

Supplement: Supplementary file 1 — Supplementary Information 1. [file 41598_2023_29606_MOESM1_ESM.zip › Attachments/alltogether_experiment/sample_hello_3dat_16_0p1_12_ifcn2_single.jpg]

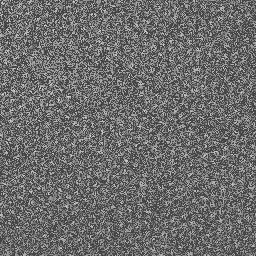

Supplement: Supplementary file 1 — Supplementary Information 1. [file 41598_2023_29606_MOESM1_ESM.zip › Attachments/alltogether_experiment/sample_hello_3dat_16_0p1_12_ori_single.jpg]

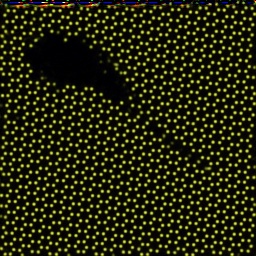

Supplement: Supplementary file 1 — Supplementary Information 1. [file 41598_2023_29606_MOESM1_ESM.zip › Attachments/alltogether_experiment/sample_hello_3dat_16_0p1_13_ganhi_single.jpg]

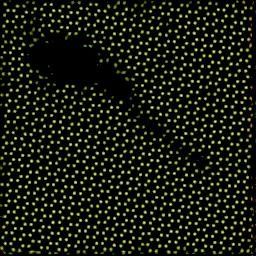

Supplement: Supplementary file 1 — Supplementary Information 1. [file 41598_2023_29606_MOESM1_ESM.zip › Attachments/alltogether_experiment/sample_hello_3dat_16_0p1_13_gan_single.jpg]

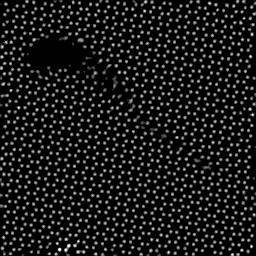

Supplement: Supplementary file 1 — Supplementary Information 1. [file 41598_2023_29606_MOESM1_ESM.zip › Attachments/alltogether_experiment/sample_hello_3dat_16_0p1_13_ifcn2_single.jpg]

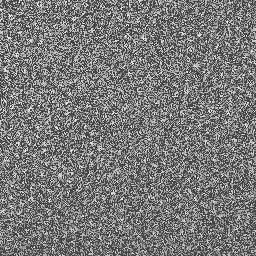

Supplement: Supplementary file 1 — Supplementary Information 1. [file 41598_2023_29606_MOESM1_ESM.zip › Attachments/alltogether_experiment/sample_hello_3dat_16_0p1_13_ori_single.jpg]

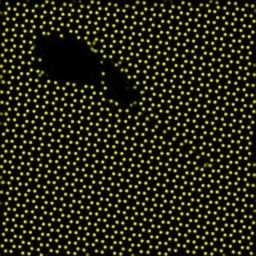

Supplement: Supplementary file 1 — Supplementary Information 1. [file 41598_2023_29606_MOESM1_ESM.zip › Attachments/alltogether_experiment/sample_hello_3dat_16_0p1_14_ganhi_single.jpg]

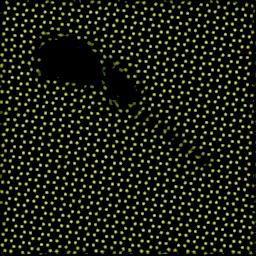

Supplement: Supplementary file 1 — Supplementary Information 1. [file 41598_2023_29606_MOESM1_ESM.zip › Attachments/alltogether_experiment/sample_hello_3dat_16_0p1_14_gan_single.jpg]

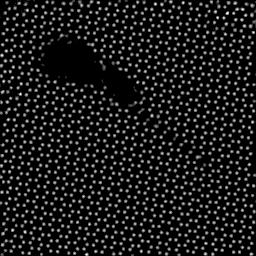

Supplement: Supplementary file 1 — Supplementary Information 1. [file 41598_2023_29606_MOESM1_ESM.zip › Attachments/alltogether_experiment/sample_hello_3dat_16_0p1_14_ifcn2_single.jpg]

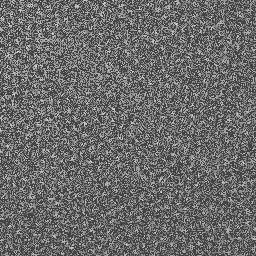

Supplement: Supplementary file 1 — Supplementary Information 1. [file 41598_2023_29606_MOESM1_ESM.zip › Attachments/alltogether_experiment/sample_hello_3dat_16_0p1_14_ori_single.jpg]

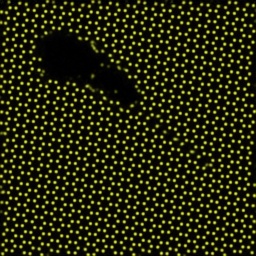

Supplement: Supplementary file 1 — Supplementary Information 1. [file 41598_2023_29606_MOESM1_ESM.zip › Attachments/alltogether_experiment/sample_hello_3dat_16_0p1_15_ganhi_single.jpg]

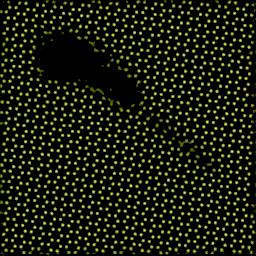

Supplement: Supplementary file 1 — Supplementary Information 1. [file 41598_2023_29606_MOESM1_ESM.zip › Attachments/alltogether_experiment/sample_hello_3dat_16_0p1_15_gan_single.jpg]

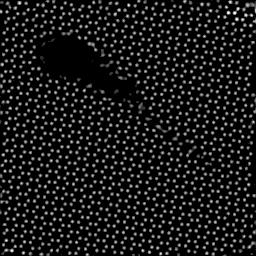

Supplement: Supplementary file 1 — Supplementary Information 1. [file 41598_2023_29606_MOESM1_ESM.zip › Attachments/alltogether_experiment/sample_hello_3dat_16_0p1_15_ifcn2_single.jpg]

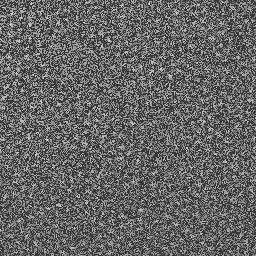

Supplement: Supplementary file 1 — Supplementary Information 1. [file 41598_2023_29606_MOESM1_ESM.zip › Attachments/alltogether_experiment/sample_hello_3dat_16_0p1_15_ori_single.jpg]

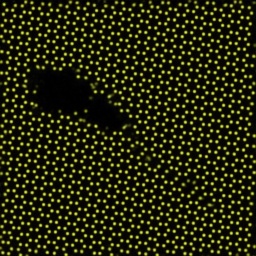

Supplement: Supplementary file 1 — Supplementary Information 1. [file 41598_2023_29606_MOESM1_ESM.zip › Attachments/alltogether_experiment/sample_hello_3dat_16_0p1_16_ganhi_single.jpg]

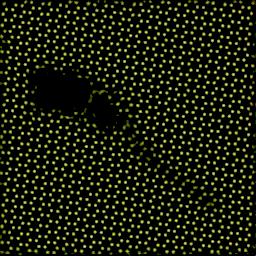

Supplement: Supplementary file 1 — Supplementary Information 1. [file 41598_2023_29606_MOESM1_ESM.zip › Attachments/alltogether_experiment/sample_hello_3dat_16_0p1_16_gan_single.jpg]

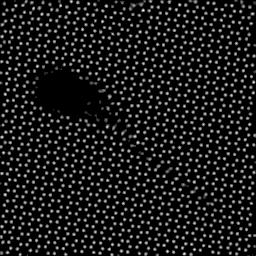

Supplement: Supplementary file 1 — Supplementary Information 1. [file 41598_2023_29606_MOESM1_ESM.zip › Attachments/alltogether_experiment/sample_hello_3dat_16_0p1_16_ifcn2_single.jpg]

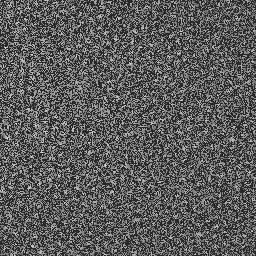

Supplement: Supplementary file 1 — Supplementary Information 1. [file 41598_2023_29606_MOESM1_ESM.zip › Attachments/alltogether_experiment/sample_hello_3dat_16_0p1_16_ori_single.jpg]

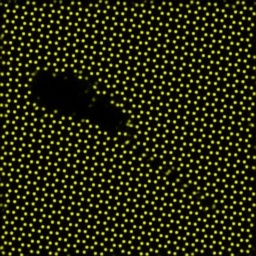

Supplement: Supplementary file 1 — Supplementary Information 1. [file 41598_2023_29606_MOESM1_ESM.zip › Attachments/alltogether_experiment/sample_hello_3dat_16_0p1_17_ganhi_single.jpg]

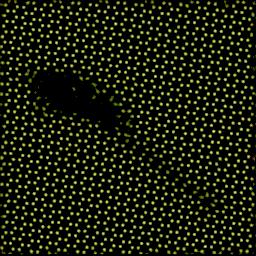

Supplement: Supplementary file 1 — Supplementary Information 1. [file 41598_2023_29606_MOESM1_ESM.zip › Attachments/alltogether_experiment/sample_hello_3dat_16_0p1_17_gan_single.jpg]

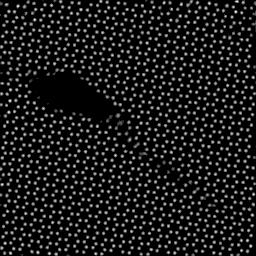

Supplement: Supplementary file 1 — Supplementary Information 1. [file 41598_2023_29606_MOESM1_ESM.zip › Attachments/alltogether_experiment/sample_hello_3dat_16_0p1_17_ifcn2_single.jpg]

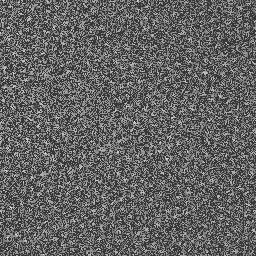

Supplement: Supplementary file 1 — Supplementary Information 1. [file 41598_2023_29606_MOESM1_ESM.zip › Attachments/alltogether_experiment/sample_hello_3dat_16_0p1_17_ori_single.jpg]

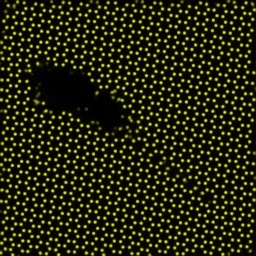

Supplement: Supplementary file 1 — Supplementary Information 1. [file 41598_2023_29606_MOESM1_ESM.zip › Attachments/alltogether_experiment/sample_hello_3dat_16_0p1_18_ganhi_single.jpg]

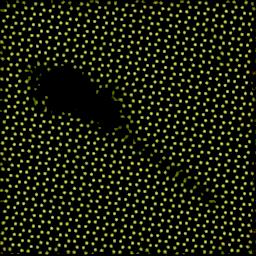

Supplement: Supplementary file 1 — Supplementary Information 1. [file 41598_2023_29606_MOESM1_ESM.zip › Attachments/alltogether_experiment/sample_hello_3dat_16_0p1_18_gan_single.jpg]

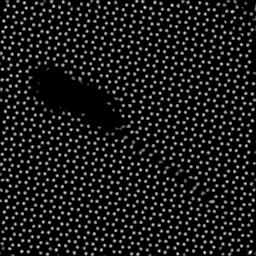

Supplement: Supplementary file 1 — Supplementary Information 1. [file 41598_2023_29606_MOESM1_ESM.zip › Attachments/alltogether_experiment/sample_hello_3dat_16_0p1_18_ifcn2_single.jpg]

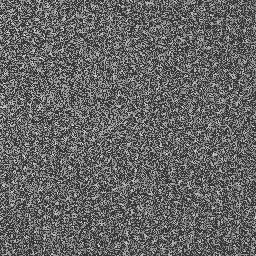

Supplement: Supplementary file 1 — Supplementary Information 1. [file 41598_2023_29606_MOESM1_ESM.zip › Attachments/alltogether_experiment/sample_hello_3dat_16_0p1_18_ori_single.jpg]

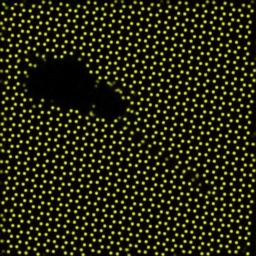

Supplement: Supplementary file 1 — Supplementary Information 1. [file 41598_2023_29606_MOESM1_ESM.zip › Attachments/alltogether_experiment/sample_hello_3dat_16_0p1_19_ganhi_single.jpg]

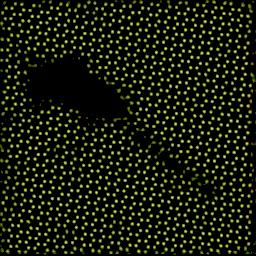

Supplement: Supplementary file 1 — Supplementary Information 1. [file 41598_2023_29606_MOESM1_ESM.zip › Attachments/alltogether_experiment/sample_hello_3dat_16_0p1_19_gan_single.jpg]

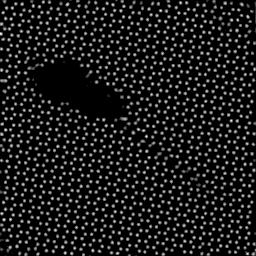

Supplement: Supplementary file 1 — Supplementary Information 1. [file 41598_2023_29606_MOESM1_ESM.zip › Attachments/alltogether_experiment/sample_hello_3dat_16_0p1_19_ifcn2_single.jpg]

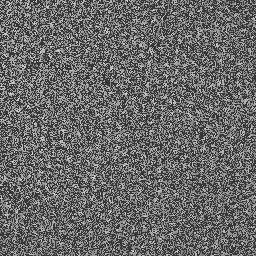

Supplement: Supplementary file 1 — Supplementary Information 1. [file 41598_2023_29606_MOESM1_ESM.zip › Attachments/alltogether_experiment/sample_hello_3dat_16_0p1_19_ori_single.jpg]

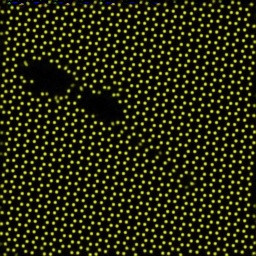

Supplement: Supplementary file 1 — Supplementary Information 1. [file 41598_2023_29606_MOESM1_ESM.zip › Attachments/alltogether_experiment/sample_hello_3dat_16_0p1_1_ganhi_single.jpg]

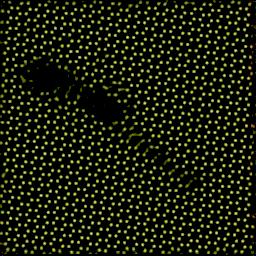

Supplement: Supplementary file 1 — Supplementary Information 1. [file 41598_2023_29606_MOESM1_ESM.zip › Attachments/alltogether_experiment/sample_hello_3dat_16_0p1_1_gan_single.jpg]

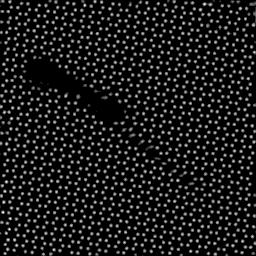

Supplement: Supplementary file 1 — Supplementary Information 1. [file 41598_2023_29606_MOESM1_ESM.zip › Attachments/alltogether_experiment/sample_hello_3dat_16_0p1_1_ifcn2_single.jpg]

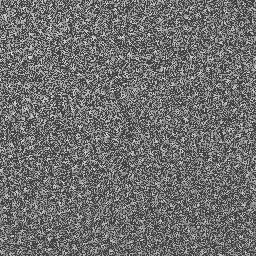

Supplement: Supplementary file 1 — Supplementary Information 1. [file 41598_2023_29606_MOESM1_ESM.zip › Attachments/alltogether_experiment/sample_hello_3dat_16_0p1_1_ori_single.jpg]

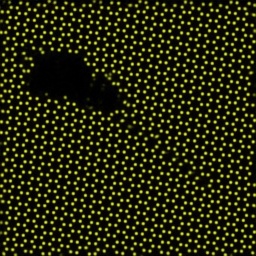

Supplement: Supplementary file 1 — Supplementary Information 1. [file 41598_2023_29606_MOESM1_ESM.zip › Attachments/alltogether_experiment/sample_hello_3dat_16_0p1_20_ganhi_single.jpg]

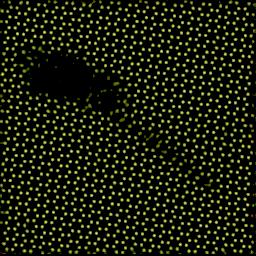

Supplement: Supplementary file 1 — Supplementary Information 1. [file 41598_2023_29606_MOESM1_ESM.zip › Attachments/alltogether_experiment/sample_hello_3dat_16_0p1_20_gan_single.jpg]

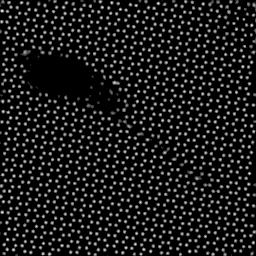

Supplement: Supplementary file 1 — Supplementary Information 1. [file 41598_2023_29606_MOESM1_ESM.zip › Attachments/alltogether_experiment/sample_hello_3dat_16_0p1_20_ifcn2_single.jpg]

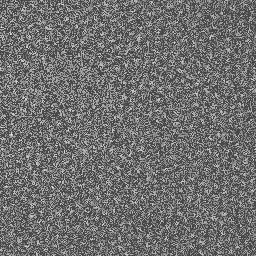

Supplement: Supplementary file 1 — Supplementary Information 1. [file 41598_2023_29606_MOESM1_ESM.zip › Attachments/alltogether_experiment/sample_hello_3dat_16_0p1_20_ori_single.jpg]

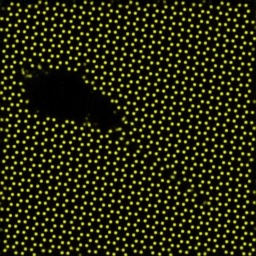

Supplement: Supplementary file 1 — Supplementary Information 1. [file 41598_2023_29606_MOESM1_ESM.zip › Attachments/alltogether_experiment/sample_hello_3dat_16_0p1_21_ganhi_single.jpg]

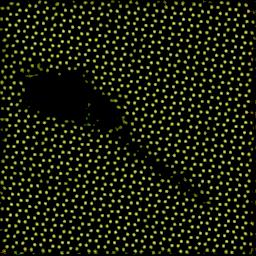

Supplement: Supplementary file 1 — Supplementary Information 1. [file 41598_2023_29606_MOESM1_ESM.zip › Attachments/alltogether_experiment/sample_hello_3dat_16_0p1_21_gan_single.jpg]

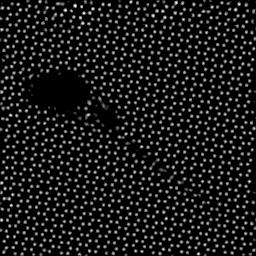

Supplement: Supplementary file 1 — Supplementary Information 1. [file 41598_2023_29606_MOESM1_ESM.zip › Attachments/alltogether_experiment/sample_hello_3dat_16_0p1_21_ifcn2_single.jpg]

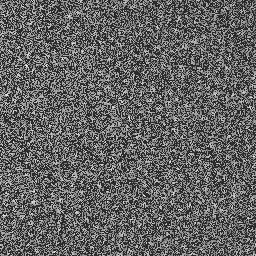

Supplement: Supplementary file 1 — Supplementary Information 1. [file 41598_2023_29606_MOESM1_ESM.zip › Attachments/alltogether_experiment/sample_hello_3dat_16_0p1_21_ori_single.jpg]

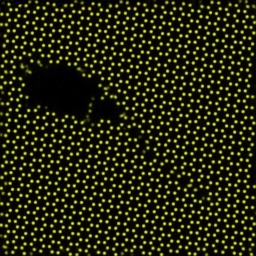

Supplement: Supplementary file 1 — Supplementary Information 1. [file 41598_2023_29606_MOESM1_ESM.zip › Attachments/alltogether_experiment/sample_hello_3dat_16_0p1_22_ganhi_single.jpg]

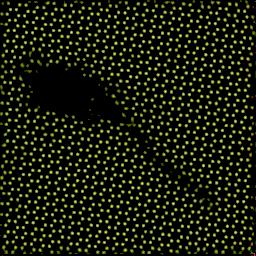

Supplement: Supplementary file 1 — Supplementary Information 1. [file 41598_2023_29606_MOESM1_ESM.zip › Attachments/alltogether_experiment/sample_hello_3dat_16_0p1_22_gan_single.jpg]

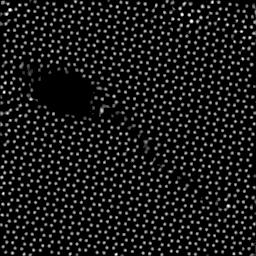

Supplement: Supplementary file 1 — Supplementary Information 1. [file 41598_2023_29606_MOESM1_ESM.zip › Attachments/alltogether_experiment/sample_hello_3dat_16_0p1_22_ifcn2_single.jpg]

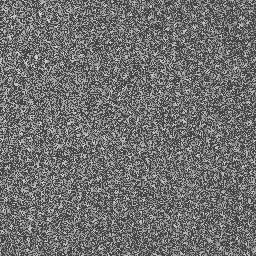

Supplement: Supplementary file 1 — Supplementary Information 1. [file 41598_2023_29606_MOESM1_ESM.zip › Attachments/alltogether_experiment/sample_hello_3dat_16_0p1_22_ori_single.jpg]

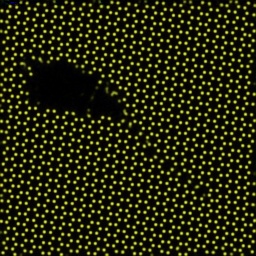

Supplement: Supplementary file 1 — Supplementary Information 1. [file 41598_2023_29606_MOESM1_ESM.zip › Attachments/alltogether_experiment/sample_hello_3dat_16_0p1_23_ganhi_single.jpg]

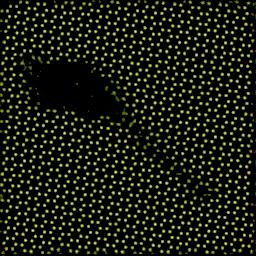

Supplement: Supplementary file 1 — Supplementary Information 1. [file 41598_2023_29606_MOESM1_ESM.zip › Attachments/alltogether_experiment/sample_hello_3dat_16_0p1_23_gan_single.jpg]

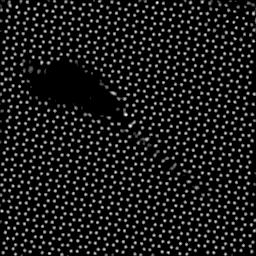

Supplement: Supplementary file 1 — Supplementary Information 1. [file 41598_2023_29606_MOESM1_ESM.zip › Attachments/alltogether_experiment/sample_hello_3dat_16_0p1_23_ifcn2_single.jpg]

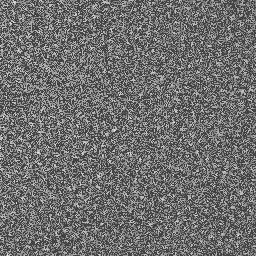

Supplement: Supplementary file 1 — Supplementary Information 1. [file 41598_2023_29606_MOESM1_ESM.zip › Attachments/alltogether_experiment/sample_hello_3dat_16_0p1_23_ori_single.jpg]

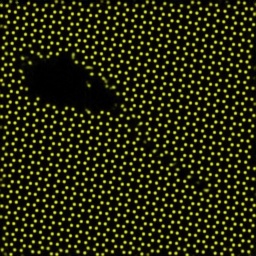

Supplement: Supplementary file 1 — Supplementary Information 1. [file 41598_2023_29606_MOESM1_ESM.zip › Attachments/alltogether_experiment/sample_hello_3dat_16_0p1_24_ganhi_single.jpg]

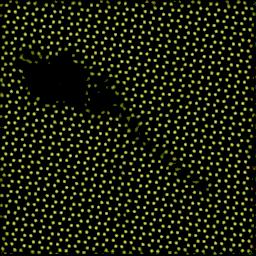

Supplement: Supplementary file 1 — Supplementary Information 1. [file 41598_2023_29606_MOESM1_ESM.zip › Attachments/alltogether_experiment/sample_hello_3dat_16_0p1_24_gan_single.jpg]

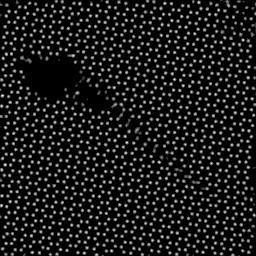

Supplement: Supplementary file 1 — Supplementary Information 1. [file 41598_2023_29606_MOESM1_ESM.zip › Attachments/alltogether_experiment/sample_hello_3dat_16_0p1_24_ifcn2_single.jpg]

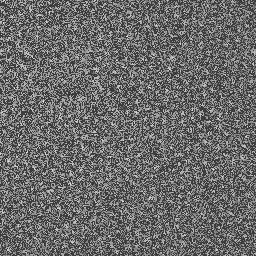

Supplement: Supplementary file 1 — Supplementary Information 1. [file 41598_2023_29606_MOESM1_ESM.zip › Attachments/alltogether_experiment/sample_hello_3dat_16_0p1_24_ori_single.jpg]

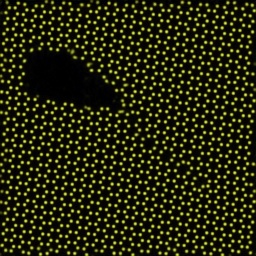

Supplement: Supplementary file 1 — Supplementary Information 1. [file 41598_2023_29606_MOESM1_ESM.zip › Attachments/alltogether_experiment/sample_hello_3dat_16_0p1_25_ganhi_single.jpg]

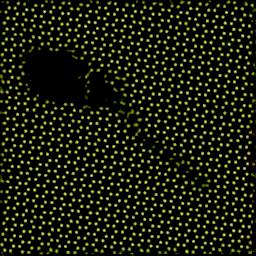

Supplement: Supplementary file 1 — Supplementary Information 1. [file 41598_2023_29606_MOESM1_ESM.zip › Attachments/alltogether_experiment/sample_hello_3dat_16_0p1_25_gan_single.jpg]

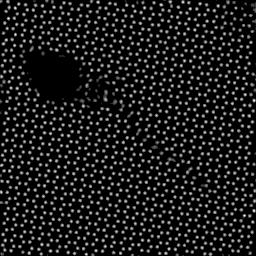

Supplement: Supplementary file 1 — Supplementary Information 1. [file 41598_2023_29606_MOESM1_ESM.zip › Attachments/alltogether_experiment/sample_hello_3dat_16_0p1_25_ifcn2_single.jpg]

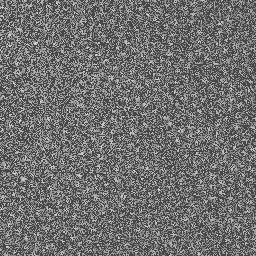

Supplement: Supplementary file 1 — Supplementary Information 1. [file 41598_2023_29606_MOESM1_ESM.zip › Attachments/alltogether_experiment/sample_hello_3dat_16_0p1_25_ori_single.jpg]

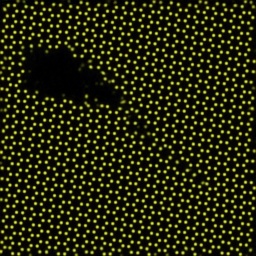

Supplement: Supplementary file 1 — Supplementary Information 1. [file 41598_2023_29606_MOESM1_ESM.zip › Attachments/alltogether_experiment/sample_hello_3dat_16_0p1_26_ganhi_single.jpg]

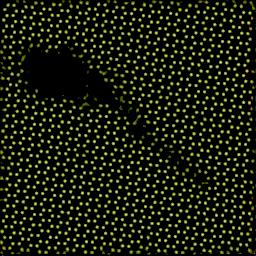

Supplement: Supplementary file 1 — Supplementary Information 1. [file 41598_2023_29606_MOESM1_ESM.zip › Attachments/alltogether_experiment/sample_hello_3dat_16_0p1_26_gan_single.jpg]

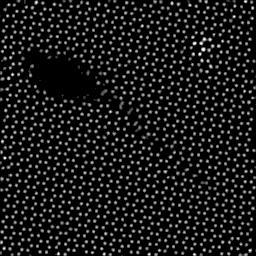

Supplement: Supplementary file 1 — Supplementary Information 1. [file 41598_2023_29606_MOESM1_ESM.zip › Attachments/alltogether_experiment/sample_hello_3dat_16_0p1_26_ifcn2_single.jpg]

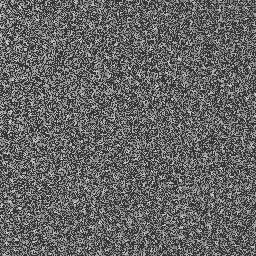

Supplement: Supplementary file 1 — Supplementary Information 1. [file 41598_2023_29606_MOESM1_ESM.zip › Attachments/alltogether_experiment/sample_hello_3dat_16_0p1_26_ori_single.jpg]

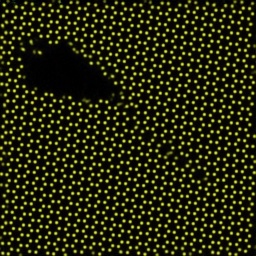

Supplement: Supplementary file 1 — Supplementary Information 1. [file 41598_2023_29606_MOESM1_ESM.zip › Attachments/alltogether_experiment/sample_hello_3dat_16_0p1_27_ganhi_single.jpg]

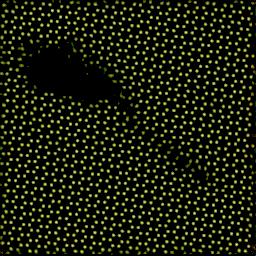

Supplement: Supplementary file 1 — Supplementary Information 1. [file 41598_2023_29606_MOESM1_ESM.zip › Attachments/alltogether_experiment/sample_hello_3dat_16_0p1_27_gan_single.jpg]

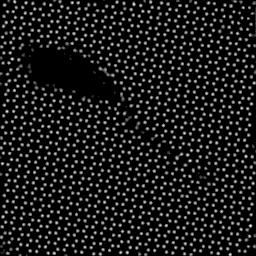

Supplement: Supplementary file 1 — Supplementary Information 1. [file 41598_2023_29606_MOESM1_ESM.zip › Attachments/alltogether_experiment/sample_hello_3dat_16_0p1_27_ifcn2_single.jpg]

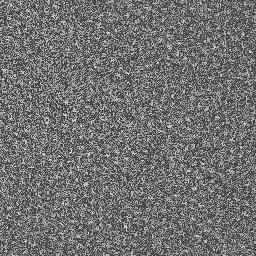

Supplement: Supplementary file 1 — Supplementary Information 1. [file 41598_2023_29606_MOESM1_ESM.zip › Attachments/alltogether_experiment/sample_hello_3dat_16_0p1_27_ori_single.jpg]

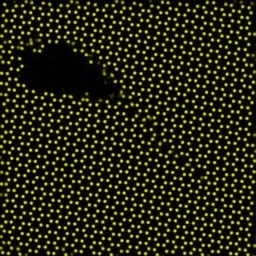

Supplement: Supplementary file 1 — Supplementary Information 1. [file 41598_2023_29606_MOESM1_ESM.zip › Attachments/alltogether_experiment/sample_hello_3dat_16_0p1_28_ganhi_single.jpg]

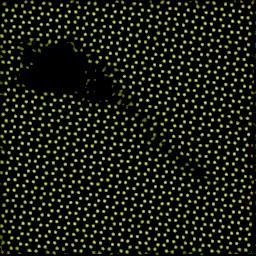

Supplement: Supplementary file 1 — Supplementary Information 1. [file 41598_2023_29606_MOESM1_ESM.zip › Attachments/alltogether_experiment/sample_hello_3dat_16_0p1_28_gan_single.jpg]

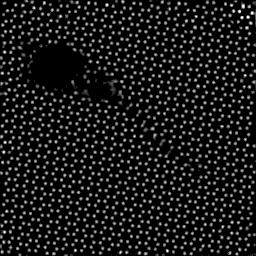

Supplement: Supplementary file 1 — Supplementary Information 1. [file 41598_2023_29606_MOESM1_ESM.zip › Attachments/alltogether_experiment/sample_hello_3dat_16_0p1_28_ifcn2_single.jpg]

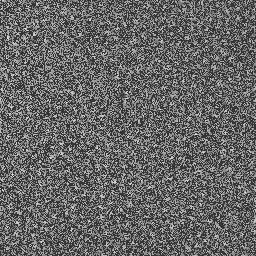

Supplement: Supplementary file 1 — Supplementary Information 1. [file 41598_2023_29606_MOESM1_ESM.zip › Attachments/alltogether_experiment/sample_hello_3dat_16_0p1_28_ori_single.jpg]

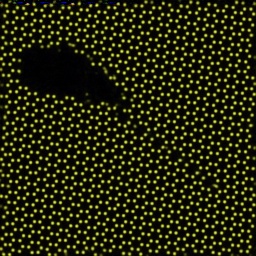

Supplement: Supplementary file 1 — Supplementary Information 1. [file 41598_2023_29606_MOESM1_ESM.zip › Attachments/alltogether_experiment/sample_hello_3dat_16_0p1_29_ganhi_single.jpg]

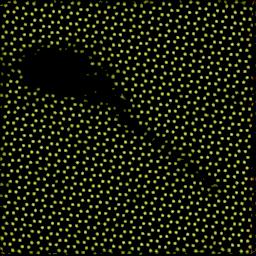

Supplement: Supplementary file 1 — Supplementary Information 1. [file 41598_2023_29606_MOESM1_ESM.zip › Attachments/alltogether_experiment/sample_hello_3dat_16_0p1_29_gan_single.jpg]

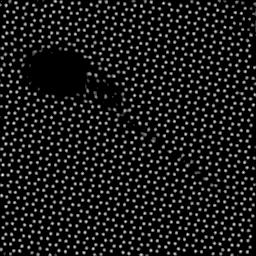

Supplement: Supplementary file 1 — Supplementary Information 1. [file 41598_2023_29606_MOESM1_ESM.zip › Attachments/alltogether_experiment/sample_hello_3dat_16_0p1_29_ifcn2_single.jpg]

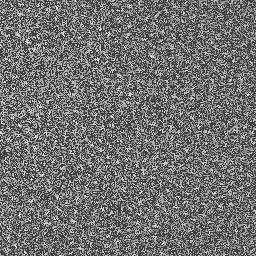

Supplement: Supplementary file 1 — Supplementary Information 1. [file 41598_2023_29606_MOESM1_ESM.zip › Attachments/alltogether_experiment/sample_hello_3dat_16_0p1_29_ori_single.jpg]

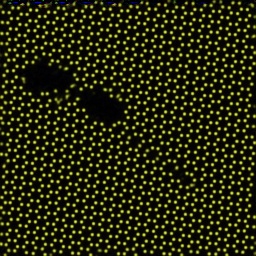

Supplement: Supplementary file 1 — Supplementary Information 1. [file 41598_2023_29606_MOESM1_ESM.zip › Attachments/alltogether_experiment/sample_hello_3dat_16_0p1_2_ganhi_single.jpg]

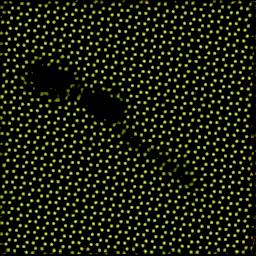

Supplement: Supplementary file 1 — Supplementary Information 1. [file 41598_2023_29606_MOESM1_ESM.zip › Attachments/alltogether_experiment/sample_hello_3dat_16_0p1_2_gan_single.jpg]

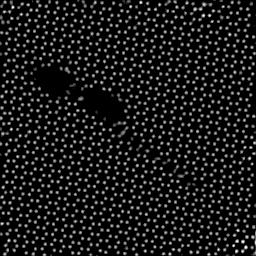

Supplement: Supplementary file 1 — Supplementary Information 1. [file 41598_2023_29606_MOESM1_ESM.zip › Attachments/alltogether_experiment/sample_hello_3dat_16_0p1_2_ifcn2_single.jpg]

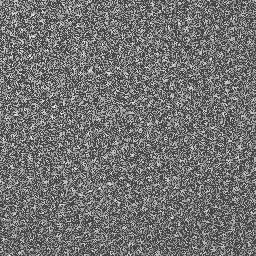

Supplement: Supplementary file 1 — Supplementary Information 1. [file 41598_2023_29606_MOESM1_ESM.zip › Attachments/alltogether_experiment/sample_hello_3dat_16_0p1_2_ori_single.jpg]

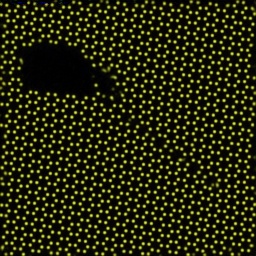

Supplement: Supplementary file 1 — Supplementary Information 1. [file 41598_2023_29606_MOESM1_ESM.zip › Attachments/alltogether_experiment/sample_hello_3dat_16_0p1_30_ganhi_single.jpg]

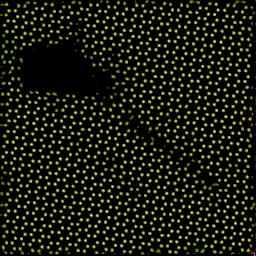

Supplement: Supplementary file 1 — Supplementary Information 1. [file 41598_2023_29606_MOESM1_ESM.zip › Attachments/alltogether_experiment/sample_hello_3dat_16_0p1_30_gan_single.jpg]

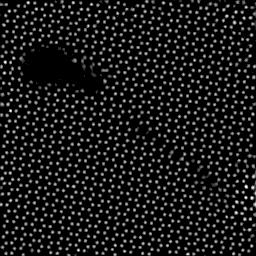

Supplement: Supplementary file 1 — Supplementary Information 1. [file 41598_2023_29606_MOESM1_ESM.zip › Attachments/alltogether_experiment/sample_hello_3dat_16_0p1_30_ifcn2_single.jpg]

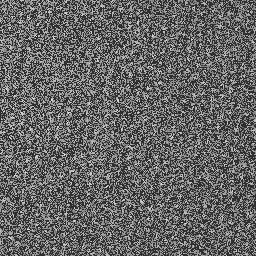

Supplement: Supplementary file 1 — Supplementary Information 1. [file 41598_2023_29606_MOESM1_ESM.zip › Attachments/alltogether_experiment/sample_hello_3dat_16_0p1_30_ori_single.jpg]

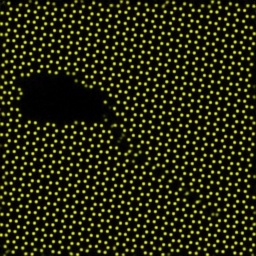

Supplement: Supplementary file 1 — Supplementary Information 1. [file 41598_2023_29606_MOESM1_ESM.zip › Attachments/alltogether_experiment/sample_hello_3dat_16_0p1_31_ganhi_single.jpg]

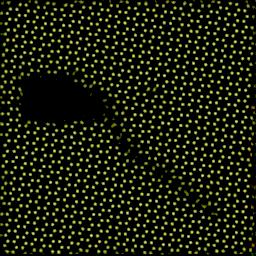

Supplement: Supplementary file 1 — Supplementary Information 1. [file 41598_2023_29606_MOESM1_ESM.zip › Attachments/alltogether_experiment/sample_hello_3dat_16_0p1_31_gan_single.jpg]

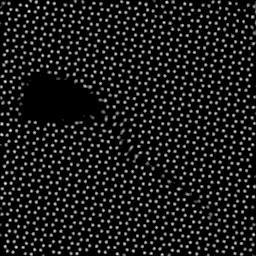

Supplement: Supplementary file 1 — Supplementary Information 1. [file 41598_2023_29606_MOESM1_ESM.zip › Attachments/alltogether_experiment/sample_hello_3dat_16_0p1_31_ifcn2_single.jpg]

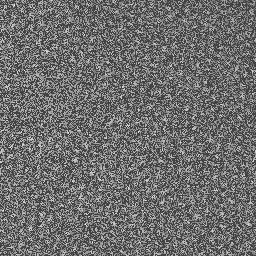

Supplement: Supplementary file 1 — Supplementary Information 1. [file 41598_2023_29606_MOESM1_ESM.zip › Attachments/alltogether_experiment/sample_hello_3dat_16_0p1_31_ori_single.jpg]

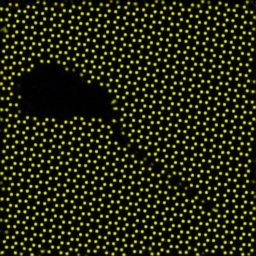

Supplement: Supplementary file 1 — Supplementary Information 1. [file 41598_2023_29606_MOESM1_ESM.zip › Attachments/alltogether_experiment/sample_hello_3dat_16_0p1_32_ganhi_single.jpg]

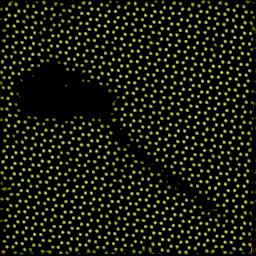

Supplement: Supplementary file 1 — Supplementary Information 1. [file 41598_2023_29606_MOESM1_ESM.zip › Attachments/alltogether_experiment/sample_hello_3dat_16_0p1_32_gan_single.jpg]

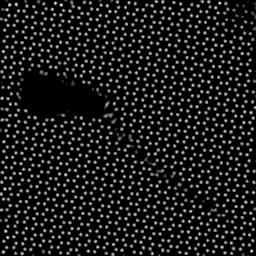

Supplement: Supplementary file 1 — Supplementary Information 1. [file 41598_2023_29606_MOESM1_ESM.zip › Attachments/alltogether_experiment/sample_hello_3dat_16_0p1_32_ifcn2_single.jpg]

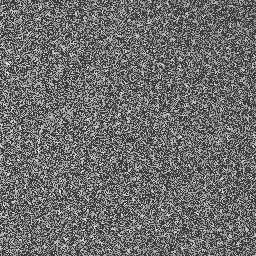

Supplement: Supplementary file 1 — Supplementary Information 1. [file 41598_2023_29606_MOESM1_ESM.zip › Attachments/alltogether_experiment/sample_hello_3dat_16_0p1_32_ori_single.jpg]

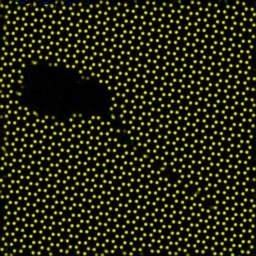

Supplement: Supplementary file 1 — Supplementary Information 1. [file 41598_2023_29606_MOESM1_ESM.zip › Attachments/alltogether_experiment/sample_hello_3dat_16_0p1_33_ganhi_single.jpg]

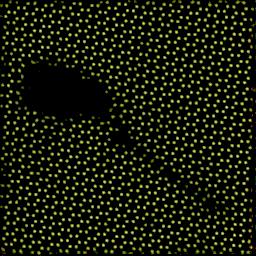

Supplement: Supplementary file 1 — Supplementary Information 1. [file 41598_2023_29606_MOESM1_ESM.zip › Attachments/alltogether_experiment/sample_hello_3dat_16_0p1_33_gan_single.jpg]

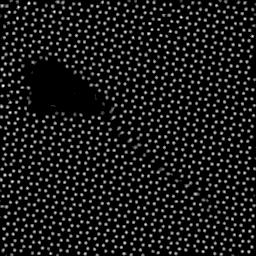

Supplement: Supplementary file 1 — Supplementary Information 1. [file 41598_2023_29606_MOESM1_ESM.zip › Attachments/alltogether_experiment/sample_hello_3dat_16_0p1_33_ifcn2_single.jpg]

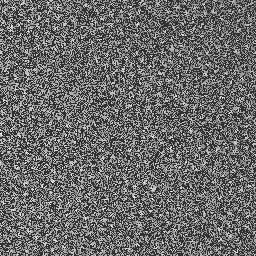

Supplement: Supplementary file 1 — Supplementary Information 1. [file 41598_2023_29606_MOESM1_ESM.zip › Attachments/alltogether_experiment/sample_hello_3dat_16_0p1_33_ori_single.jpg]
