# Supplementary figures and images for: A small-dataset-trained deep learning framework for identifying atoms on transmission electron microscopy images (part 1 of 2)
Source: Sci Rep. 2023 Feb 14;13:2631. doi: 10.1038/s41598-023-29606-9 (PMC9929221; doi:10.1038/s41598-023-29606-9)

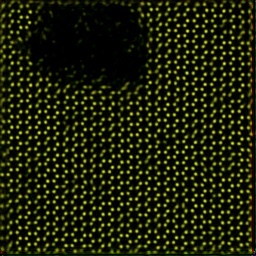

Supplement: Supplementary file 1 — Supplementary Information 1. [file 41598_2023_29606_MOESM1_ESM.zip › Attachments/alltogether_experiment/experimental_image_crop_experimental_image_1-10_gan.jpg]

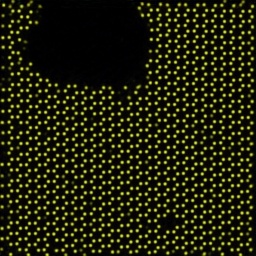

Supplement: Supplementary file 1 — Supplementary Information 1. [file 41598_2023_29606_MOESM1_ESM.zip › Attachments/alltogether_experiment/experimental_image_crop_experimental_image_1-10_ganhi.jpg]

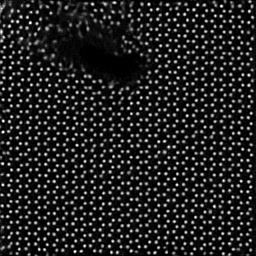

Supplement: Supplementary file 1 — Supplementary Information 1. [file 41598_2023_29606_MOESM1_ESM.zip › Attachments/alltogether_experiment/experimental_image_crop_experimental_image_1-10_ifcn2.jpg]

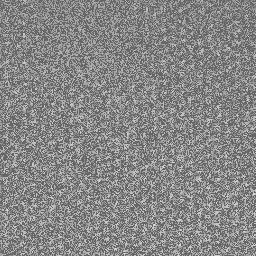

Supplement: Supplementary file 1 — Supplementary Information 1. [file 41598_2023_29606_MOESM1_ESM.zip › Attachments/alltogether_experiment/experimental_image_crop_experimental_image_1-10_ori.jpg]

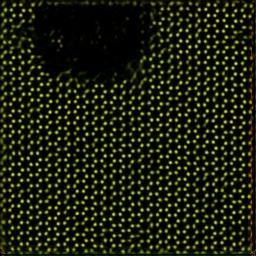

Supplement: Supplementary file 1 — Supplementary Information 1. [file 41598_2023_29606_MOESM1_ESM.zip › Attachments/alltogether_experiment/experimental_image_crop_experimental_image_1-15_gan.jpg]

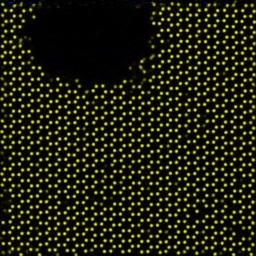

Supplement: Supplementary file 1 — Supplementary Information 1. [file 41598_2023_29606_MOESM1_ESM.zip › Attachments/alltogether_experiment/experimental_image_crop_experimental_image_1-15_ganhi.jpg]

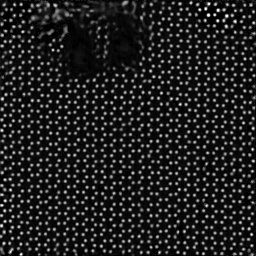

Supplement: Supplementary file 1 — Supplementary Information 1. [file 41598_2023_29606_MOESM1_ESM.zip › Attachments/alltogether_experiment/experimental_image_crop_experimental_image_1-15_ifcn2.jpg]

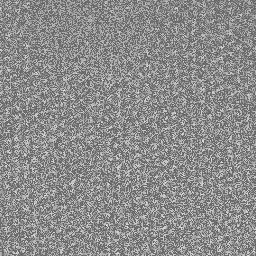

Supplement: Supplementary file 1 — Supplementary Information 1. [file 41598_2023_29606_MOESM1_ESM.zip › Attachments/alltogether_experiment/experimental_image_crop_experimental_image_1-15_ori.jpg]

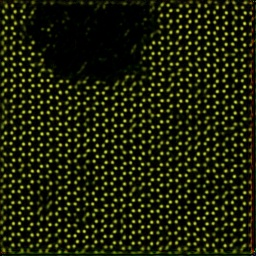

Supplement: Supplementary file 1 — Supplementary Information 1. [file 41598_2023_29606_MOESM1_ESM.zip › Attachments/alltogether_experiment/experimental_image_crop_experimental_image_1-19_gan.jpg]

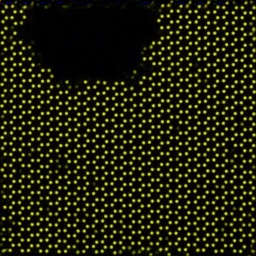

Supplement: Supplementary file 1 — Supplementary Information 1. [file 41598_2023_29606_MOESM1_ESM.zip › Attachments/alltogether_experiment/experimental_image_crop_experimental_image_1-19_ganhi.jpg]

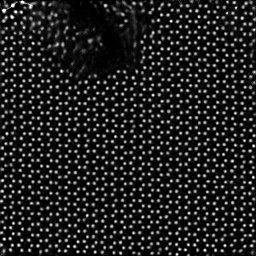

Supplement: Supplementary file 1 — Supplementary Information 1. [file 41598_2023_29606_MOESM1_ESM.zip › Attachments/alltogether_experiment/experimental_image_crop_experimental_image_1-19_ifcn2.jpg]

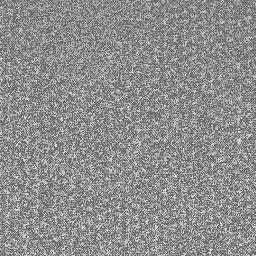

Supplement: Supplementary file 1 — Supplementary Information 1. [file 41598_2023_29606_MOESM1_ESM.zip › Attachments/alltogether_experiment/experimental_image_crop_experimental_image_1-19_ori.jpg]

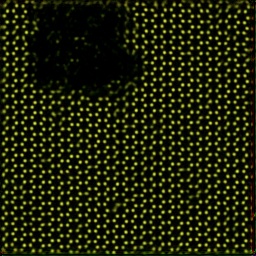

Supplement: Supplementary file 1 — Supplementary Information 1. [file 41598_2023_29606_MOESM1_ESM.zip › Attachments/alltogether_experiment/experimental_image_crop_experimental_image_1-1_gan.jpg]

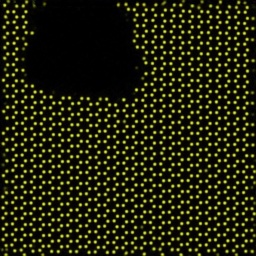

Supplement: Supplementary file 1 — Supplementary Information 1. [file 41598_2023_29606_MOESM1_ESM.zip › Attachments/alltogether_experiment/experimental_image_crop_experimental_image_1-1_ganhi.jpg]

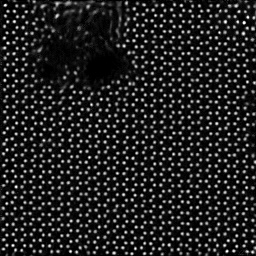

Supplement: Supplementary file 1 — Supplementary Information 1. [file 41598_2023_29606_MOESM1_ESM.zip › Attachments/alltogether_experiment/experimental_image_crop_experimental_image_1-1_ifcn2.jpg]

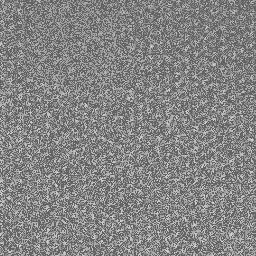

Supplement: Supplementary file 1 — Supplementary Information 1. [file 41598_2023_29606_MOESM1_ESM.zip › Attachments/alltogether_experiment/experimental_image_crop_experimental_image_1-1_ori.jpg]

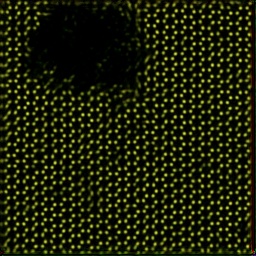

Supplement: Supplementary file 1 — Supplementary Information 1. [file 41598_2023_29606_MOESM1_ESM.zip › Attachments/alltogether_experiment/experimental_image_crop_experimental_image_1-5_gan.jpg]

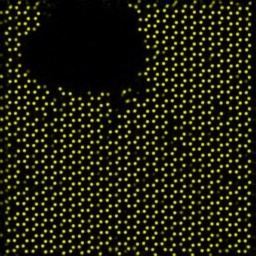

Supplement: Supplementary file 1 — Supplementary Information 1. [file 41598_2023_29606_MOESM1_ESM.zip › Attachments/alltogether_experiment/experimental_image_crop_experimental_image_1-5_ganhi.jpg]

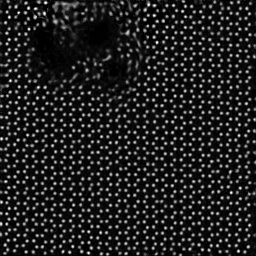

Supplement: Supplementary file 1 — Supplementary Information 1. [file 41598_2023_29606_MOESM1_ESM.zip › Attachments/alltogether_experiment/experimental_image_crop_experimental_image_1-5_ifcn2.jpg]

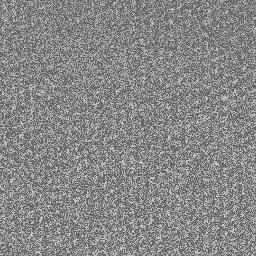

Supplement: Supplementary file 1 — Supplementary Information 1. [file 41598_2023_29606_MOESM1_ESM.zip › Attachments/alltogether_experiment/experimental_image_crop_experimental_image_1-5_ori.jpg]

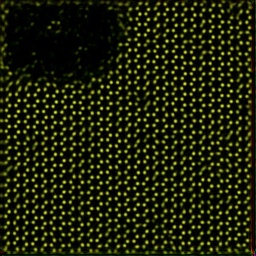

Supplement: Supplementary file 1 — Supplementary Information 1. [file 41598_2023_29606_MOESM1_ESM.zip › Attachments/alltogether_experiment/experimental_image_crop_experimental_image_2-10_gan.jpg]

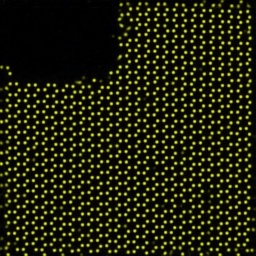

Supplement: Supplementary file 1 — Supplementary Information 1. [file 41598_2023_29606_MOESM1_ESM.zip › Attachments/alltogether_experiment/experimental_image_crop_experimental_image_2-10_ganhi.jpg]

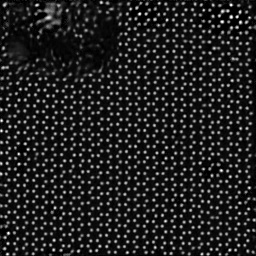

Supplement: Supplementary file 1 — Supplementary Information 1. [file 41598_2023_29606_MOESM1_ESM.zip › Attachments/alltogether_experiment/experimental_image_crop_experimental_image_2-10_ifcn2.jpg]

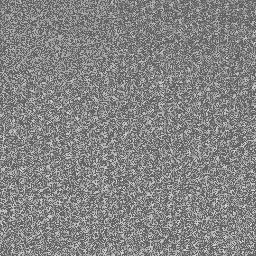

Supplement: Supplementary file 1 — Supplementary Information 1. [file 41598_2023_29606_MOESM1_ESM.zip › Attachments/alltogether_experiment/experimental_image_crop_experimental_image_2-10_ori.jpg]

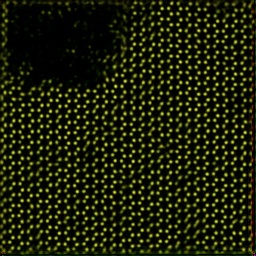

Supplement: Supplementary file 1 — Supplementary Information 1. [file 41598_2023_29606_MOESM1_ESM.zip › Attachments/alltogether_experiment/experimental_image_crop_experimental_image_2-15_gan.jpg]

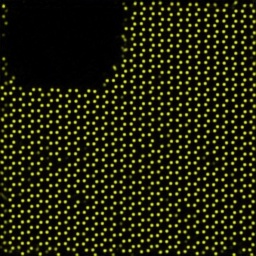

Supplement: Supplementary file 1 — Supplementary Information 1. [file 41598_2023_29606_MOESM1_ESM.zip › Attachments/alltogether_experiment/experimental_image_crop_experimental_image_2-15_ganhi.jpg]

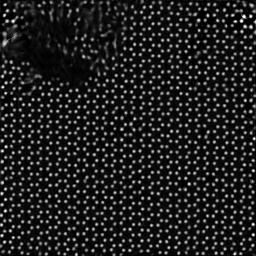

Supplement: Supplementary file 1 — Supplementary Information 1. [file 41598_2023_29606_MOESM1_ESM.zip › Attachments/alltogether_experiment/experimental_image_crop_experimental_image_2-15_ifcn2.jpg]

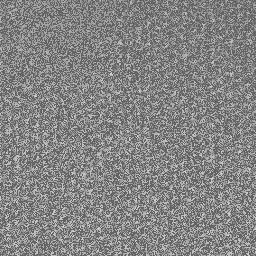

Supplement: Supplementary file 1 — Supplementary Information 1. [file 41598_2023_29606_MOESM1_ESM.zip › Attachments/alltogether_experiment/experimental_image_crop_experimental_image_2-15_ori.jpg]

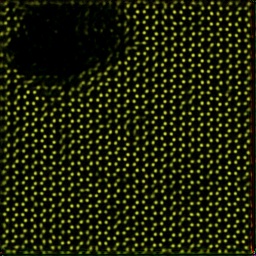

Supplement: Supplementary file 1 — Supplementary Information 1. [file 41598_2023_29606_MOESM1_ESM.zip › Attachments/alltogether_experiment/experimental_image_crop_experimental_image_2-19_gan.jpg]

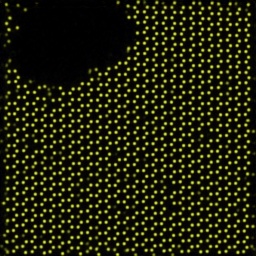

Supplement: Supplementary file 1 — Supplementary Information 1. [file 41598_2023_29606_MOESM1_ESM.zip › Attachments/alltogether_experiment/experimental_image_crop_experimental_image_2-19_ganhi.jpg]

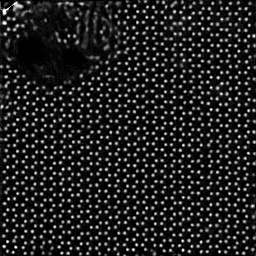

Supplement: Supplementary file 1 — Supplementary Information 1. [file 41598_2023_29606_MOESM1_ESM.zip › Attachments/alltogether_experiment/experimental_image_crop_experimental_image_2-19_ifcn2.jpg]

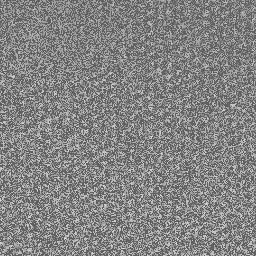

Supplement: Supplementary file 1 — Supplementary Information 1. [file 41598_2023_29606_MOESM1_ESM.zip › Attachments/alltogether_experiment/experimental_image_crop_experimental_image_2-19_ori.jpg]

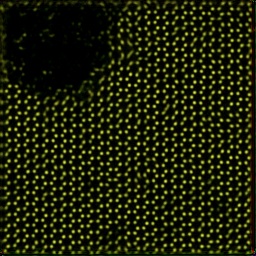

Supplement: Supplementary file 1 — Supplementary Information 1. [file 41598_2023_29606_MOESM1_ESM.zip › Attachments/alltogether_experiment/experimental_image_crop_experimental_image_2-1_gan.jpg]

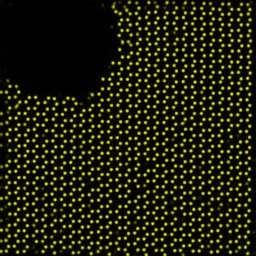

Supplement: Supplementary file 1 — Supplementary Information 1. [file 41598_2023_29606_MOESM1_ESM.zip › Attachments/alltogether_experiment/experimental_image_crop_experimental_image_2-1_ganhi.jpg]

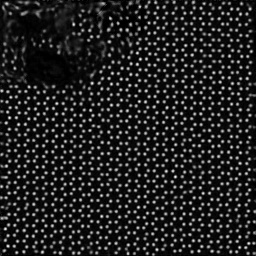

Supplement: Supplementary file 1 — Supplementary Information 1. [file 41598_2023_29606_MOESM1_ESM.zip › Attachments/alltogether_experiment/experimental_image_crop_experimental_image_2-1_ifcn2.jpg]

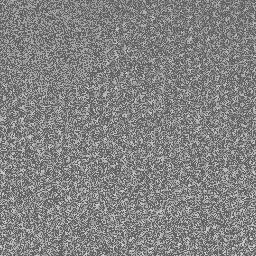

Supplement: Supplementary file 1 — Supplementary Information 1. [file 41598_2023_29606_MOESM1_ESM.zip › Attachments/alltogether_experiment/experimental_image_crop_experimental_image_2-1_ori.jpg]

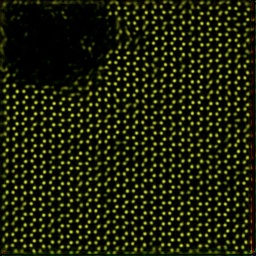

Supplement: Supplementary file 1 — Supplementary Information 1. [file 41598_2023_29606_MOESM1_ESM.zip › Attachments/alltogether_experiment/experimental_image_crop_experimental_image_2-5_gan.jpg]

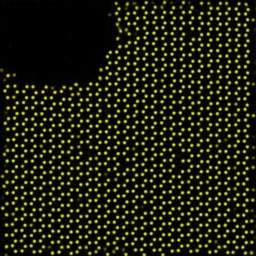

Supplement: Supplementary file 1 — Supplementary Information 1. [file 41598_2023_29606_MOESM1_ESM.zip › Attachments/alltogether_experiment/experimental_image_crop_experimental_image_2-5_ganhi.jpg]

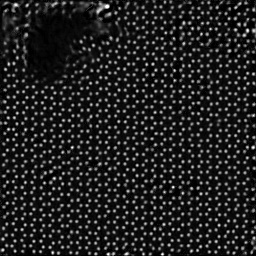

Supplement: Supplementary file 1 — Supplementary Information 1. [file 41598_2023_29606_MOESM1_ESM.zip › Attachments/alltogether_experiment/experimental_image_crop_experimental_image_2-5_ifcn2.jpg]

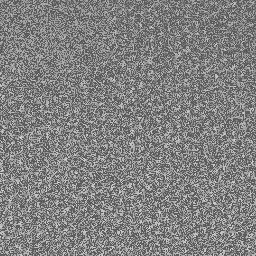

Supplement: Supplementary file 1 — Supplementary Information 1. [file 41598_2023_29606_MOESM1_ESM.zip › Attachments/alltogether_experiment/experimental_image_crop_experimental_image_2-5_ori.jpg]

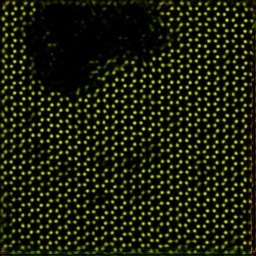

Supplement: Supplementary file 1 — Supplementary Information 1. [file 41598_2023_29606_MOESM1_ESM.zip › Attachments/alltogether_experiment/experimental_image_crop_experimental_image_3_19_gan.jpg]

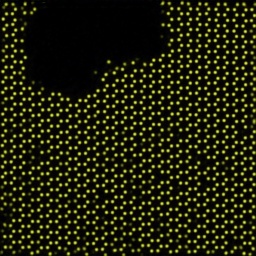

Supplement: Supplementary file 1 — Supplementary Information 1. [file 41598_2023_29606_MOESM1_ESM.zip › Attachments/alltogether_experiment/experimental_image_crop_experimental_image_3_19_ganhi.jpg]

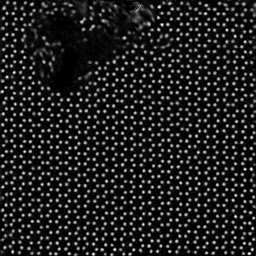

Supplement: Supplementary file 1 — Supplementary Information 1. [file 41598_2023_29606_MOESM1_ESM.zip › Attachments/alltogether_experiment/experimental_image_crop_experimental_image_3_19_ifcn2.jpg]

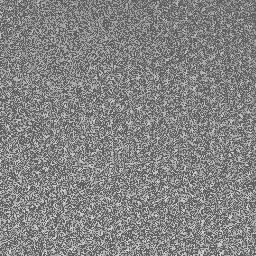

Supplement: Supplementary file 1 — Supplementary Information 1. [file 41598_2023_29606_MOESM1_ESM.zip › Attachments/alltogether_experiment/experimental_image_crop_experimental_image_3_19_ori.jpg]

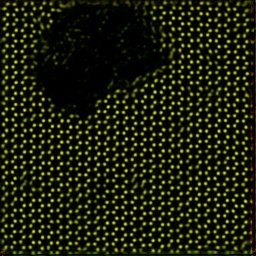

Supplement: Supplementary file 1 — Supplementary Information 1. [file 41598_2023_29606_MOESM1_ESM.zip › Attachments/alltogether_experiment/experimental_image_crop_experimental_image_3_1_gan.jpg]

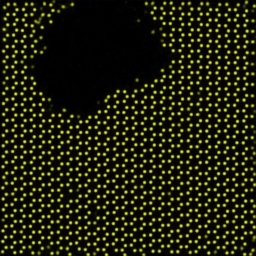

Supplement: Supplementary file 1 — Supplementary Information 1. [file 41598_2023_29606_MOESM1_ESM.zip › Attachments/alltogether_experiment/experimental_image_crop_experimental_image_3_1_ganhi.jpg]

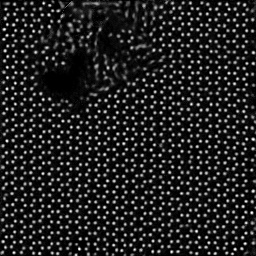

Supplement: Supplementary file 1 — Supplementary Information 1. [file 41598_2023_29606_MOESM1_ESM.zip › Attachments/alltogether_experiment/experimental_image_crop_experimental_image_3_1_ifcn2.jpg]

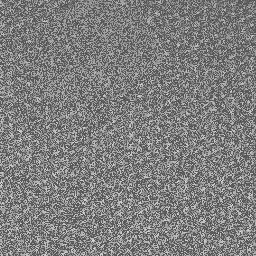

Supplement: Supplementary file 1 — Supplementary Information 1. [file 41598_2023_29606_MOESM1_ESM.zip › Attachments/alltogether_experiment/experimental_image_crop_experimental_image_3_1_ori.jpg]

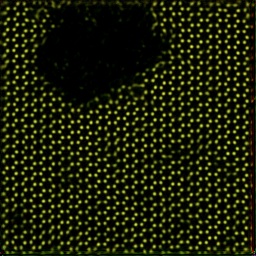

Supplement: Supplementary file 1 — Supplementary Information 1. [file 41598_2023_29606_MOESM1_ESM.zip › Attachments/alltogether_experiment/experimental_image_crop_experimental_image_3_5_gan.jpg]

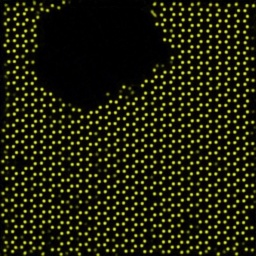

Supplement: Supplementary file 1 — Supplementary Information 1. [file 41598_2023_29606_MOESM1_ESM.zip › Attachments/alltogether_experiment/experimental_image_crop_experimental_image_3_5_ganhi.jpg]

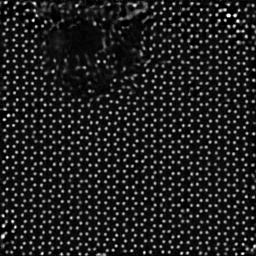

Supplement: Supplementary file 1 — Supplementary Information 1. [file 41598_2023_29606_MOESM1_ESM.zip › Attachments/alltogether_experiment/experimental_image_crop_experimental_image_3_5_ifcn2.jpg]

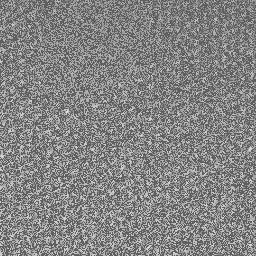

Supplement: Supplementary file 1 — Supplementary Information 1. [file 41598_2023_29606_MOESM1_ESM.zip › Attachments/alltogether_experiment/experimental_image_crop_experimental_image_3_5_ori.jpg]

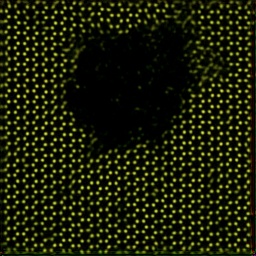

Supplement: Supplementary file 1 — Supplementary Information 1. [file 41598_2023_29606_MOESM1_ESM.zip › Attachments/alltogether_experiment/experimental_image_crop_experimental_image_4-10_gan.jpg]

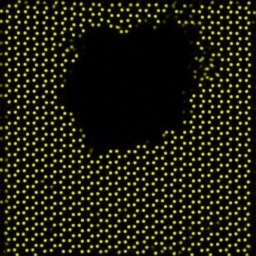

Supplement: Supplementary file 1 — Supplementary Information 1. [file 41598_2023_29606_MOESM1_ESM.zip › Attachments/alltogether_experiment/experimental_image_crop_experimental_image_4-10_ganhi.jpg]

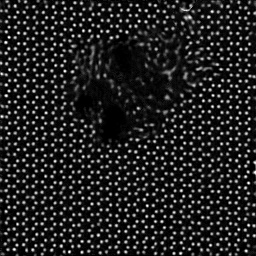

Supplement: Supplementary file 1 — Supplementary Information 1. [file 41598_2023_29606_MOESM1_ESM.zip › Attachments/alltogether_experiment/experimental_image_crop_experimental_image_4-10_ifcn2.jpg]

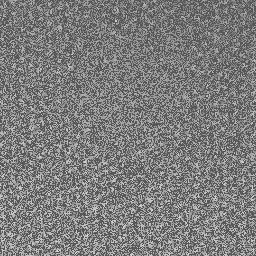

Supplement: Supplementary file 1 — Supplementary Information 1. [file 41598_2023_29606_MOESM1_ESM.zip › Attachments/alltogether_experiment/experimental_image_crop_experimental_image_4-10_ori.jpg]

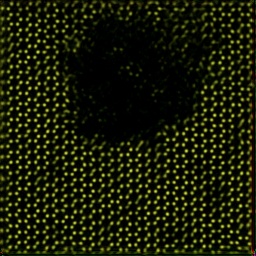

Supplement: Supplementary file 1 — Supplementary Information 1. [file 41598_2023_29606_MOESM1_ESM.zip › Attachments/alltogether_experiment/experimental_image_crop_experimental_image_4-15_gan.jpg]

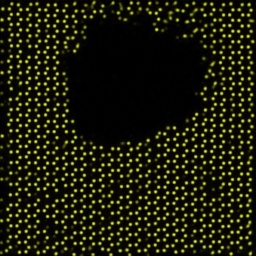

Supplement: Supplementary file 1 — Supplementary Information 1. [file 41598_2023_29606_MOESM1_ESM.zip › Attachments/alltogether_experiment/experimental_image_crop_experimental_image_4-15_ganhi.jpg]

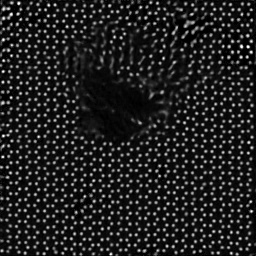

Supplement: Supplementary file 1 — Supplementary Information 1. [file 41598_2023_29606_MOESM1_ESM.zip › Attachments/alltogether_experiment/experimental_image_crop_experimental_image_4-15_ifcn2.jpg]

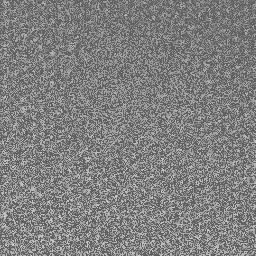

Supplement: Supplementary file 1 — Supplementary Information 1. [file 41598_2023_29606_MOESM1_ESM.zip › Attachments/alltogether_experiment/experimental_image_crop_experimental_image_4-15_ori.jpg]

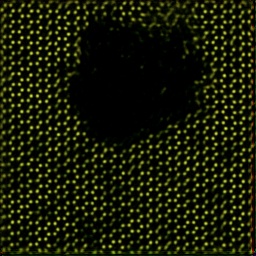

Supplement: Supplementary file 1 — Supplementary Information 1. [file 41598_2023_29606_MOESM1_ESM.zip › Attachments/alltogether_experiment/experimental_image_crop_experimental_image_4-19_gan.jpg]

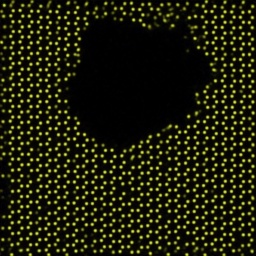

Supplement: Supplementary file 1 — Supplementary Information 1. [file 41598_2023_29606_MOESM1_ESM.zip › Attachments/alltogether_experiment/experimental_image_crop_experimental_image_4-19_ganhi.jpg]

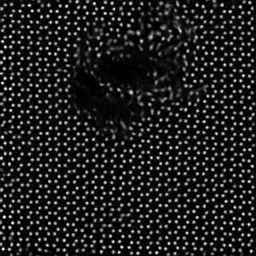

Supplement: Supplementary file 1 — Supplementary Information 1. [file 41598_2023_29606_MOESM1_ESM.zip › Attachments/alltogether_experiment/experimental_image_crop_experimental_image_4-19_ifcn2.jpg]

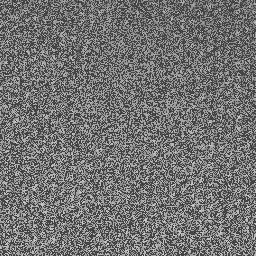

Supplement: Supplementary file 1 — Supplementary Information 1. [file 41598_2023_29606_MOESM1_ESM.zip › Attachments/alltogether_experiment/experimental_image_crop_experimental_image_4-19_ori.jpg]

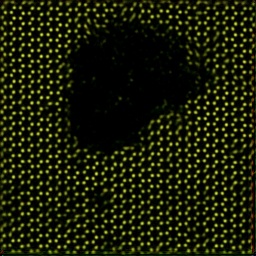

Supplement: Supplementary file 1 — Supplementary Information 1. [file 41598_2023_29606_MOESM1_ESM.zip › Attachments/alltogether_experiment/experimental_image_crop_experimental_image_4-1_gan.jpg]

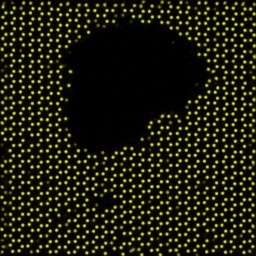

Supplement: Supplementary file 1 — Supplementary Information 1. [file 41598_2023_29606_MOESM1_ESM.zip › Attachments/alltogether_experiment/experimental_image_crop_experimental_image_4-1_ganhi.jpg]

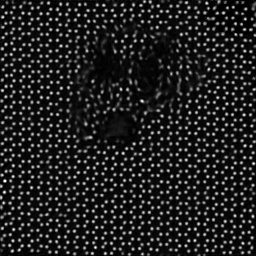

Supplement: Supplementary file 1 — Supplementary Information 1. [file 41598_2023_29606_MOESM1_ESM.zip › Attachments/alltogether_experiment/experimental_image_crop_experimental_image_4-1_ifcn2.jpg]

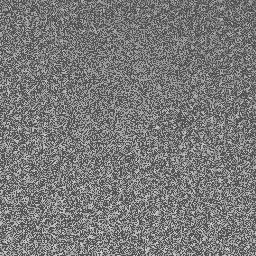

Supplement: Supplementary file 1 — Supplementary Information 1. [file 41598_2023_29606_MOESM1_ESM.zip › Attachments/alltogether_experiment/experimental_image_crop_experimental_image_4-1_ori.jpg]

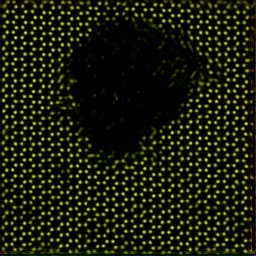

Supplement: Supplementary file 1 — Supplementary Information 1. [file 41598_2023_29606_MOESM1_ESM.zip › Attachments/alltogether_experiment/experimental_image_crop_experimental_image_4-5_gan.jpg]

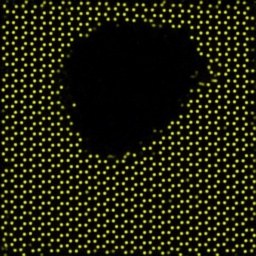

Supplement: Supplementary file 1 — Supplementary Information 1. [file 41598_2023_29606_MOESM1_ESM.zip › Attachments/alltogether_experiment/experimental_image_crop_experimental_image_4-5_ganhi.jpg]

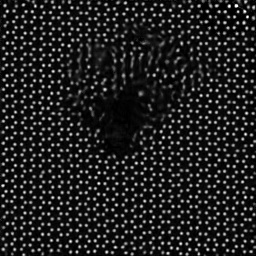

Supplement: Supplementary file 1 — Supplementary Information 1. [file 41598_2023_29606_MOESM1_ESM.zip › Attachments/alltogether_experiment/experimental_image_crop_experimental_image_4-5_ifcn2.jpg]

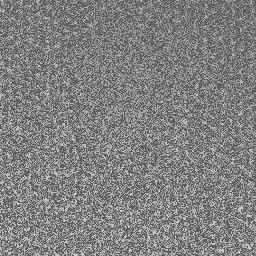

Supplement: Supplementary file 1 — Supplementary Information 1. [file 41598_2023_29606_MOESM1_ESM.zip › Attachments/alltogether_experiment/experimental_image_crop_experimental_image_4-5_ori.jpg]

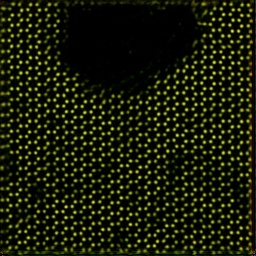

Supplement: Supplementary file 1 — Supplementary Information 1. [file 41598_2023_29606_MOESM1_ESM.zip › Attachments/alltogether_experiment/experimental_image_crop_experimental_image_5-10_gan.jpg]

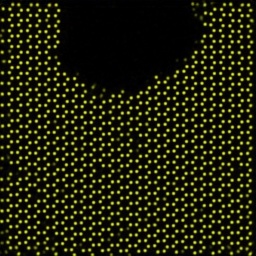

Supplement: Supplementary file 1 — Supplementary Information 1. [file 41598_2023_29606_MOESM1_ESM.zip › Attachments/alltogether_experiment/experimental_image_crop_experimental_image_5-10_ganhi.jpg]

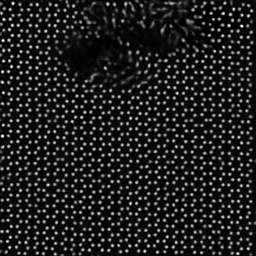

Supplement: Supplementary file 1 — Supplementary Information 1. [file 41598_2023_29606_MOESM1_ESM.zip › Attachments/alltogether_experiment/experimental_image_crop_experimental_image_5-10_ifcn2.jpg]

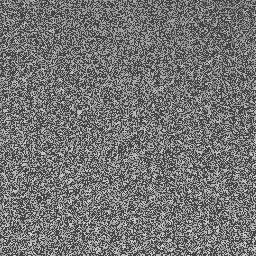

Supplement: Supplementary file 1 — Supplementary Information 1. [file 41598_2023_29606_MOESM1_ESM.zip › Attachments/alltogether_experiment/experimental_image_crop_experimental_image_5-10_ori.jpg]

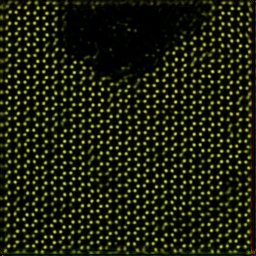

Supplement: Supplementary file 1 — Supplementary Information 1. [file 41598_2023_29606_MOESM1_ESM.zip › Attachments/alltogether_experiment/experimental_image_crop_experimental_image_5-15_gan.jpg]

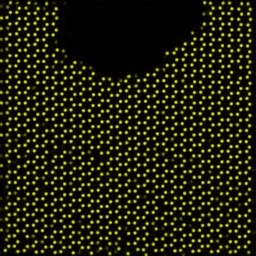

Supplement: Supplementary file 1 — Supplementary Information 1. [file 41598_2023_29606_MOESM1_ESM.zip › Attachments/alltogether_experiment/experimental_image_crop_experimental_image_5-15_ganhi.jpg]

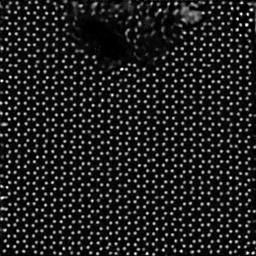

Supplement: Supplementary file 1 — Supplementary Information 1. [file 41598_2023_29606_MOESM1_ESM.zip › Attachments/alltogether_experiment/experimental_image_crop_experimental_image_5-15_ifcn2.jpg]

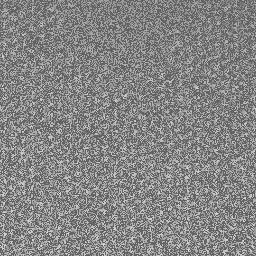

Supplement: Supplementary file 1 — Supplementary Information 1. [file 41598_2023_29606_MOESM1_ESM.zip › Attachments/alltogether_experiment/experimental_image_crop_experimental_image_5-15_ori.jpg]

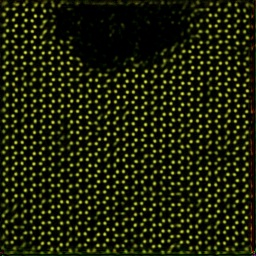

Supplement: Supplementary file 1 — Supplementary Information 1. [file 41598_2023_29606_MOESM1_ESM.zip › Attachments/alltogether_experiment/experimental_image_crop_experimental_image_5-19_gan.jpg]

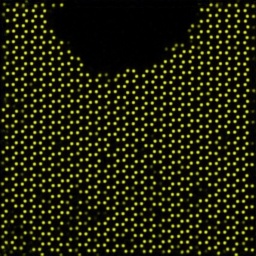

Supplement: Supplementary file 1 — Supplementary Information 1. [file 41598_2023_29606_MOESM1_ESM.zip › Attachments/alltogether_experiment/experimental_image_crop_experimental_image_5-19_ganhi.jpg]

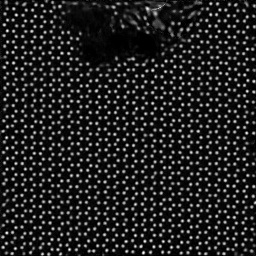

Supplement: Supplementary file 1 — Supplementary Information 1. [file 41598_2023_29606_MOESM1_ESM.zip › Attachments/alltogether_experiment/experimental_image_crop_experimental_image_5-19_ifcn2.jpg]

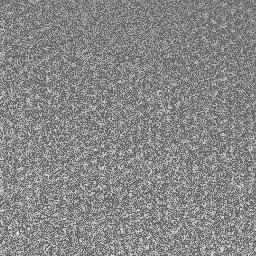

Supplement: Supplementary file 1 — Supplementary Information 1. [file 41598_2023_29606_MOESM1_ESM.zip › Attachments/alltogether_experiment/experimental_image_crop_experimental_image_5-19_ori.jpg]

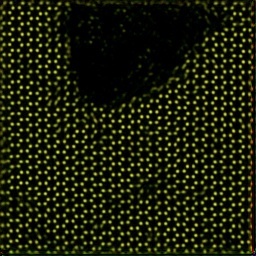

Supplement: Supplementary file 1 — Supplementary Information 1. [file 41598_2023_29606_MOESM1_ESM.zip › Attachments/alltogether_experiment/experimental_image_crop_experimental_image_5-1_gan.jpg]

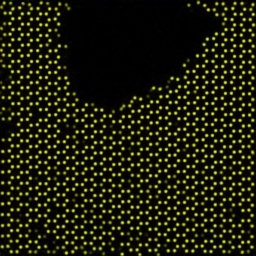

Supplement: Supplementary file 1 — Supplementary Information 1. [file 41598_2023_29606_MOESM1_ESM.zip › Attachments/alltogether_experiment/experimental_image_crop_experimental_image_5-1_ganhi.jpg]

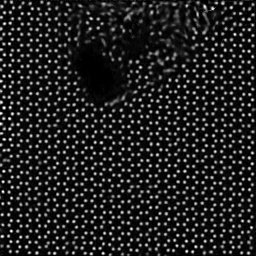

Supplement: Supplementary file 1 — Supplementary Information 1. [file 41598_2023_29606_MOESM1_ESM.zip › Attachments/alltogether_experiment/experimental_image_crop_experimental_image_5-1_ifcn2.jpg]

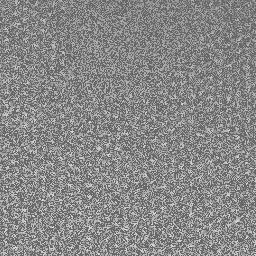

Supplement: Supplementary file 1 — Supplementary Information 1. [file 41598_2023_29606_MOESM1_ESM.zip › Attachments/alltogether_experiment/experimental_image_crop_experimental_image_5-1_ori.jpg]

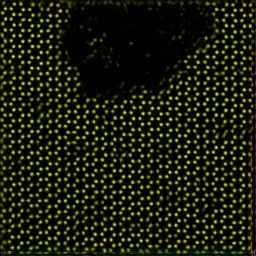

Supplement: Supplementary file 1 — Supplementary Information 1. [file 41598_2023_29606_MOESM1_ESM.zip › Attachments/alltogether_experiment/experimental_image_crop_experimental_image_5-5_gan.jpg]

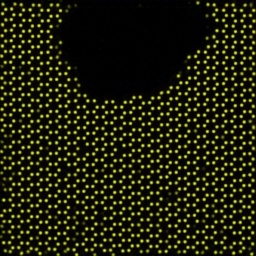

Supplement: Supplementary file 1 — Supplementary Information 1. [file 41598_2023_29606_MOESM1_ESM.zip › Attachments/alltogether_experiment/experimental_image_crop_experimental_image_5-5_ganhi.jpg]

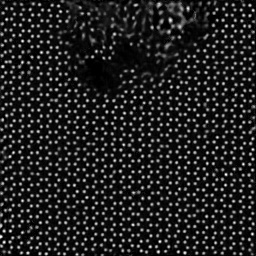

Supplement: Supplementary file 1 — Supplementary Information 1. [file 41598_2023_29606_MOESM1_ESM.zip › Attachments/alltogether_experiment/experimental_image_crop_experimental_image_5-5_ifcn2.jpg]

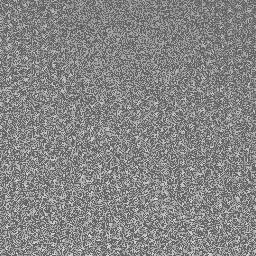

Supplement: Supplementary file 1 — Supplementary Information 1. [file 41598_2023_29606_MOESM1_ESM.zip › Attachments/alltogether_experiment/experimental_image_crop_experimental_image_5-5_ori.jpg]

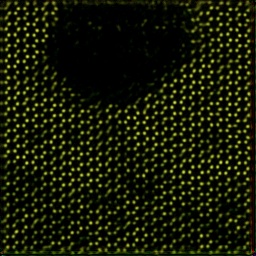

Supplement: Supplementary file 1 — Supplementary Information 1. [file 41598_2023_29606_MOESM1_ESM.zip › Attachments/alltogether_experiment/experimental_image_crop_experimental_image_6-1_gan.jpg]

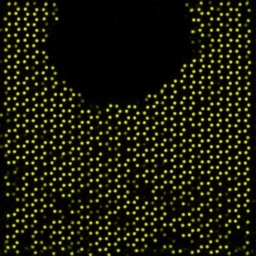

Supplement: Supplementary file 1 — Supplementary Information 1. [file 41598_2023_29606_MOESM1_ESM.zip › Attachments/alltogether_experiment/experimental_image_crop_experimental_image_6-1_ganhi.jpg]

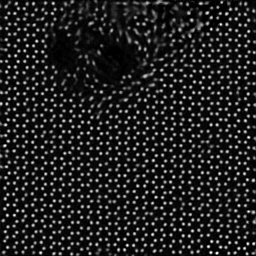

Supplement: Supplementary file 1 — Supplementary Information 1. [file 41598_2023_29606_MOESM1_ESM.zip › Attachments/alltogether_experiment/experimental_image_crop_experimental_image_6-1_ifcn2.jpg]

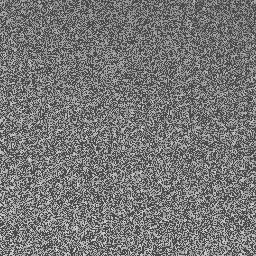

Supplement: Supplementary file 1 — Supplementary Information 1. [file 41598_2023_29606_MOESM1_ESM.zip › Attachments/alltogether_experiment/experimental_image_crop_experimental_image_6-1_ori.jpg]

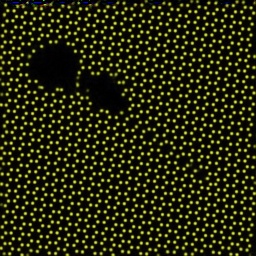

Supplement: Supplementary file 1 — Supplementary Information 1. [file 41598_2023_29606_MOESM1_ESM.zip › Attachments/alltogether_experiment/sample_hello_3dat_16_0p1_10_ganhi_single.jpg]

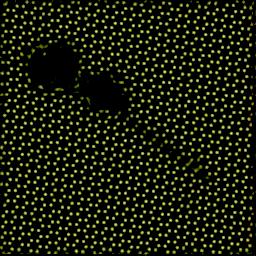

Supplement: Supplementary file 1 — Supplementary Information 1. [file 41598_2023_29606_MOESM1_ESM.zip › Attachments/alltogether_experiment/sample_hello_3dat_16_0p1_10_gan_single.jpg]

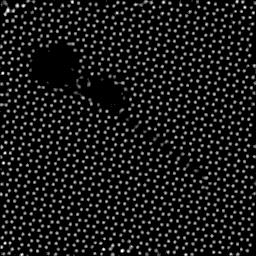

Supplement: Supplementary file 1 — Supplementary Information 1. [file 41598_2023_29606_MOESM1_ESM.zip › Attachments/alltogether_experiment/sample_hello_3dat_16_0p1_10_ifcn2_single.jpg]

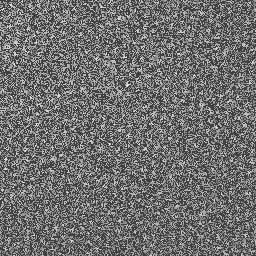

Supplement: Supplementary file 1 — Supplementary Information 1. [file 41598_2023_29606_MOESM1_ESM.zip › Attachments/alltogether_experiment/sample_hello_3dat_16_0p1_10_ori_single.jpg]
