# Supplementary material for: A small-dataset-trained deep learning framework for identifying atoms on transmission electron microscopy images
Source: Sci Rep. 2023 Feb 14;13:2631. doi: 10.1038/s41598-023-29606-9 (PMC9929221; doi:10.1038/s41598-023-29606-9)
Supplement: Supplementary file 2 — Supplementary Information 2. [file 41598_2023_29606_MOESM2_ESM.pdf]

Details of the attachments

First of all, the ‘Attachments.zip’ contains three folders: ‘alltogether_experiment’, ‘alltogether_simulation’ and ‘altogether_detail_compare’. Each of the three folders is described below.

The folder ‘alltogether_experiment’ contains 64 sets of experimental images, one of which is shown in Figure 1. Figure 1(a) is named ‘experimental_image_crop_Experimental_image_1-1_ori.jpg’. Figure 1(b) is named ‘experimental_image_crop_experimental_image_1-1_ganhi.jpg’. Figure 1(c) is named ‘experimental_image_crop_experimental_image_1-1_gan.jpg’. Figure 1(d) is named ‘experimental_image_crop_experimental_image_1-1_ifcn2.jpg’.

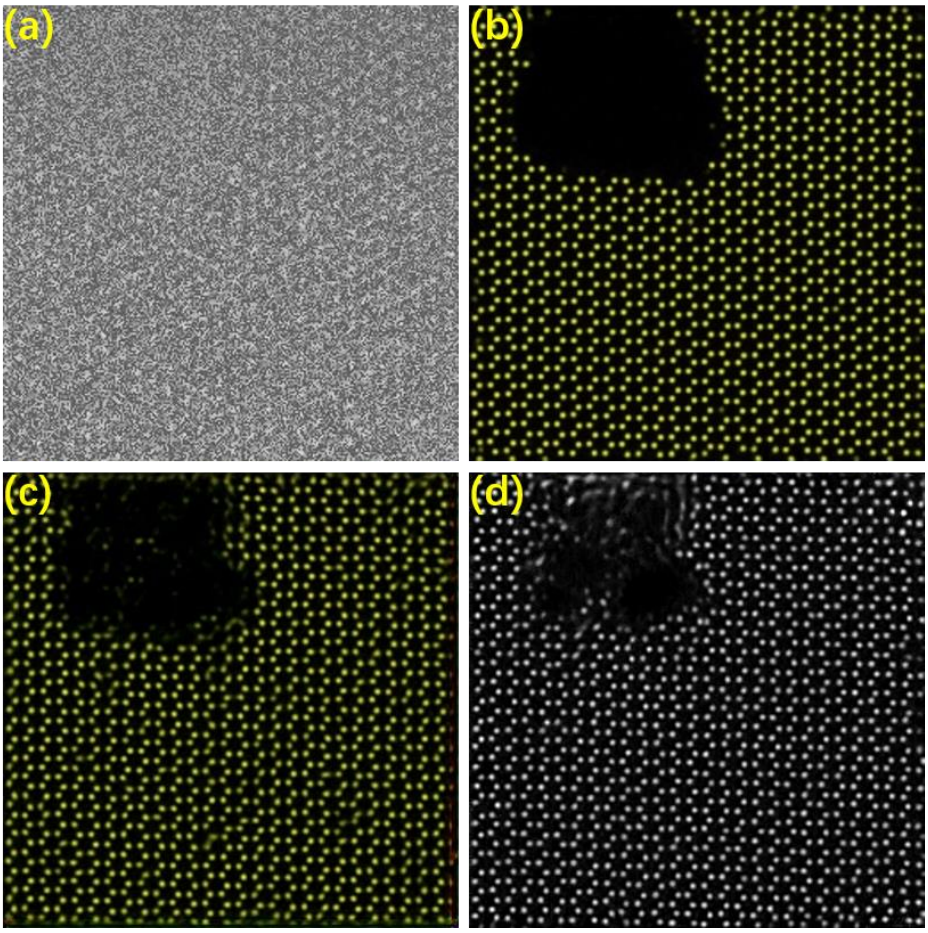

Figure 1. Results of a set of experimental images (a) The imitated low-dose image with 70% pixels were reserved is simulated from the experimental image. (b) The probability map of atoms at each pixel predicted from normal-dose experimental image via using AP-GANs. (c) The probability map of atoms at each pixel predicted from (a) via using AP-GANs. (d) The probability map of atoms at each pixel predicted from (a) via using FCNs2.

The folder 'alltogether_simulation' contains 24 sets of simulated images, one of which is shown in Figure 2. Figure 2(a) is named 'a_simulation_image_40_13_ori.jpg'. Figure 2(b) is named 'a_simulation_image_40_13_ganhi.jpg'. Figure 2(c) is named 'a_simulation_image_40_13_gan.jpg'. Figure 2(d) is named

‘a_simulation_image_40_13_ifcn2.jpg’.

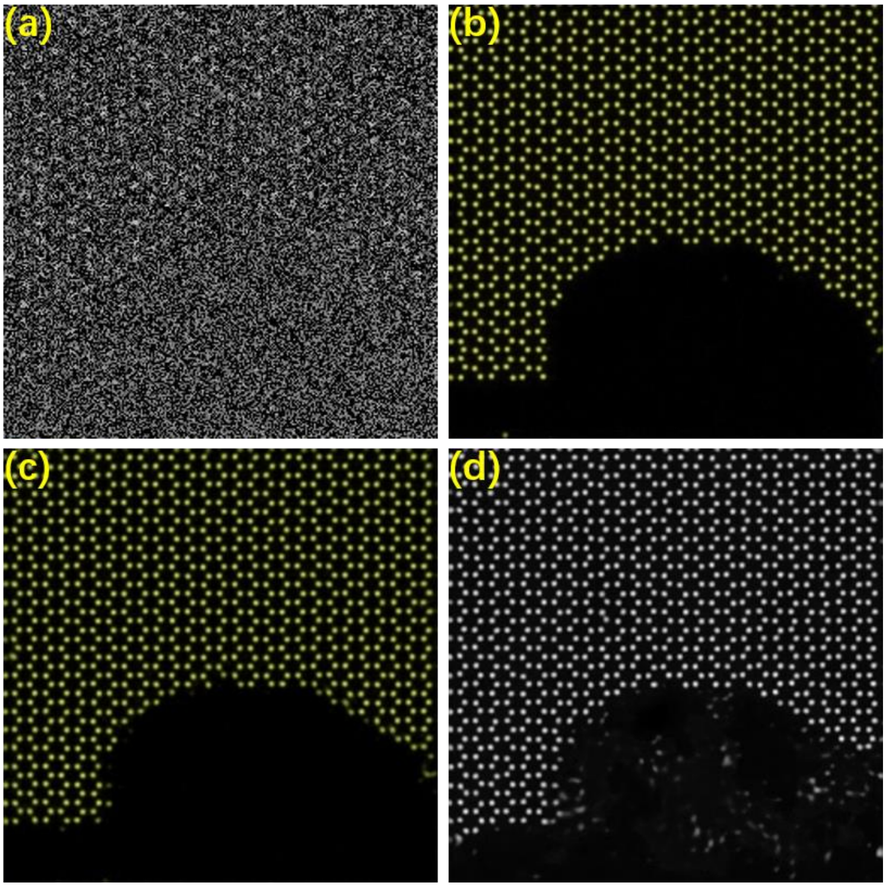

Figure 2. Results of a set of simulated images (a) The imitated low-dose image with 30% pixels were reserved is simulated from the simulated image. (b) The probability map of atoms at each pixel predicted from normal-dose simulated image via using AP-GANs. (c) The probability map of atoms at each pixel predicted from (a) via using AP-GANs. (d) The probability map of atoms at each pixel predicted from (a) via using FCNs2.

The folder ‘alltogether_simulation’ contains 2 sets of experimental images and 2 sets of simulated images. The green crosses highlight clear artefacts that might not exist in

the vacuum.

In conclusion, artefacts on prediction via using FCNs2 may be more serious on vacuum, which were illustrated for simulated and experimental images in Attachments.
