# Supplementary material for: A small-dataset-trained deep learning framework for identifying atoms on transmission electron microscopy images
Source: Sci Rep. 2023 Feb 14;13:2631. doi: 10.1038/s41598-023-29606-9 (PMC9929221; doi:10.1038/s41598-023-29606-9)
Supplement: Supplementary file 3 — Supplementary Information 3. [file 41598_2023_29606_MOESM3_ESM.pdf]

Supplementary Information

A small-dataset-trained deep learning framework for identifying atoms on transmission electron microscopy images

Yuan Chen[a], Shangpeng Liu[a], Peiran Tong[b], Ying Huang[a], He Tian[b], Fang Lin[a,*]

a College of Electronic Engineering, South China Agricultural University, Guangzhou, Guangdong 510642, China

b State Key Laboratory of Silicon Materials, School of Materials Science and Engineering, Zhejiang University, Hangzhou, Zhejiang 310027, China

*** Corresponding author. E-mail address: linfang@scau.edu.cn (F. Lin)**

The loss function of generator is composed of three parts with different weights, which can be written as

$$L_G = \lambda_{adv} \cdot L_{adv}\big(D(y), D(\hat{y})\big) + \lambda_{ssim} \cdot L_{ssim}(y, \hat{y}) + \lambda_{psnr} \cdot L_{psnr}(y, \hat{y}) \quad (1)$$

where L_{adv} is the adversarial loss of generator and discriminator, L_{ssim} is the loss of the structural similarity (SSIM), L_{psnr} is the loss of the peak signal-to-noise ratio (PSNR), and λ_{adv}, λ_{ssim} and λ_{psnr} are their weights, respectively.

In Eq. 1. L_{adv} is yield as

$$L_{adv} = L1_{loss}(y_i, \hat{y}_i) \quad (2)$$

where $L1_{loss}$ represent Mean Absolute Error (MAE), i is the index of the pixel, and y_i and $\hat{y}_i$ are the output of the network and the ground truth at the ith pixel, respectively. The L_{ssim} is given by

$$L_{ssim} = 1 - SSIM(y_i, \hat{y}_i) \quad (3)$$

where $SSIM(y_i, \hat{y}_i)$ is to calculate the structural similarity between the output of the

network and the ground truth. And L_{psnr} is given by

$$L_{psnr} = PSNR(y_i, \hat{y}_i) \tag{4}$$

where y_i and $\hat{y}_i$ are the output of the network and the ground truth at the ith pixel, respectively. What's more, $PSNR$ is defined as

$$PSNR = 10 \cdot \log_{10}\left(\frac{MAX_I^2}{MSE}\right) \tag{5}$$

where MAX_I is the maximum intensity on image and MSE is the Mean Squared Error of raw image and processed image.

The loss function of discriminator is the Mean Squared Error (MSE) of the ground truth and the output of the network, which can be written as

$$L_D = L_{MSE}\big(D(y), D(\hat{y})\big) \tag{6}$$

where $D(y)$ and $D(\hat{y})$ are the output of the discriminator, and y and $\hat{y}$ are the output of the network and the ground truth.

The L_{MSE} in Eq. 6 is simply:

$$L_{MSE} = \frac{1}{n}\sum_{i=1}^{n}(y_i - \hat{y}_i)^2 \tag{7}$$

where n is the number of pixels on image and y_i and $\hat{y}_i$ are the ground truth and the output of the network at the ith pixel, respectively.

**Name of parameters**	
Learning rate	0.0002
The weight of adversarial loss	0.5
The weight of loss of the structural similarity (SSIM)	-1
The weight of loss of the peak signal-to-noise ratio (PSNR)	-0.1

Supplementary Table 1. Hyperparameters of training networks.

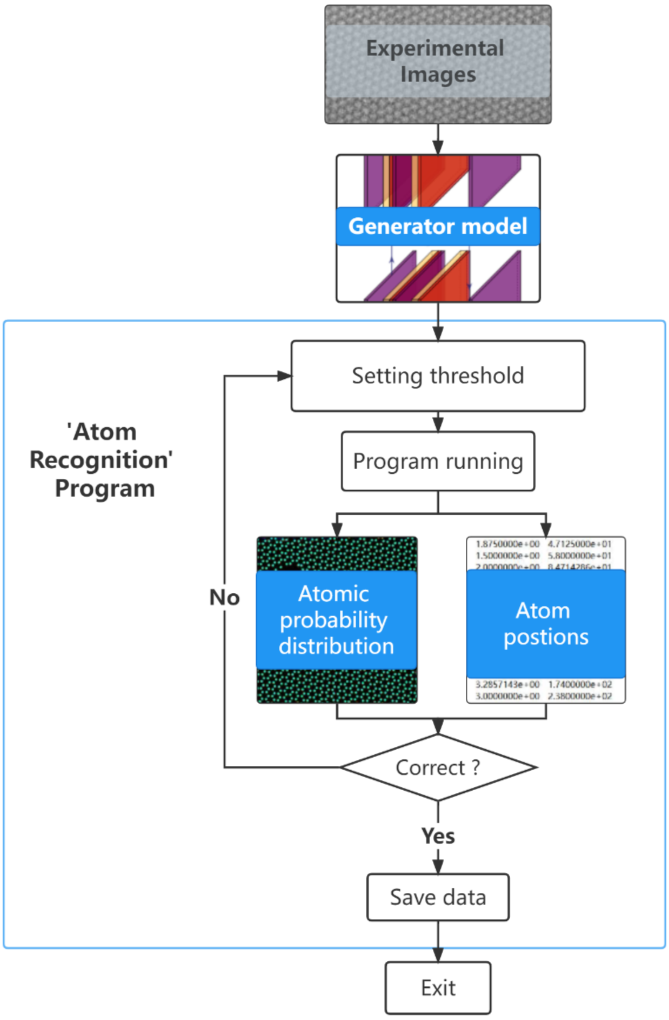

Supplementary Figure 1. Flowchart of the Atom Recognition Program (ARP). Firstly, two thresholds are set: the intensity threshold and the threshold of the area occupied by a single atom. Then, all pixels exceeding the intensity threshold and projecting along at least two directions (such as X and Y directions) are classified to measure the center of each classification, which is the round center of each atom.

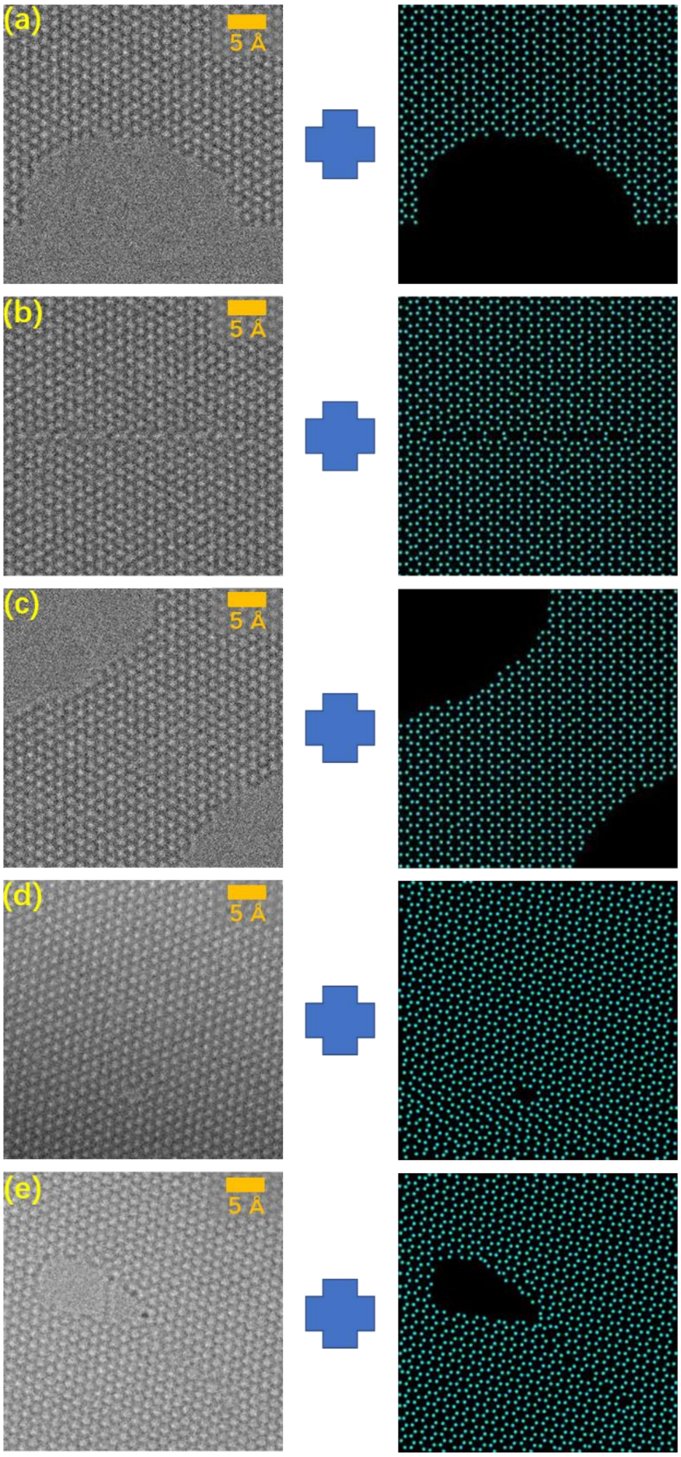

Supplementary Figure 2. Input-output pairs containing line defects and irregular boundaries in training set. (a-c) Simulated and (d-e) experimental pairs. Images in the first column is the input raw images and the second column are their outputs.

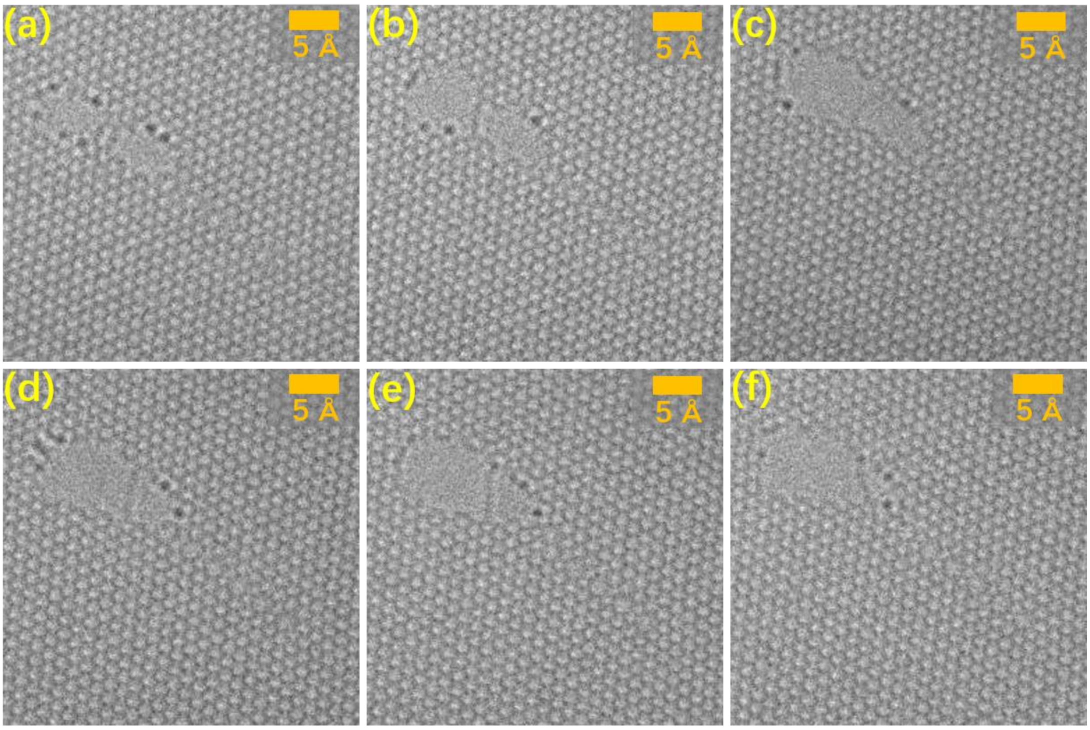

Supplementary Figure 3. The raw experimental images of structural evolutions. Figures from (a) to (f) correspond to those from (k) to (p) of Fig.2 in main text.

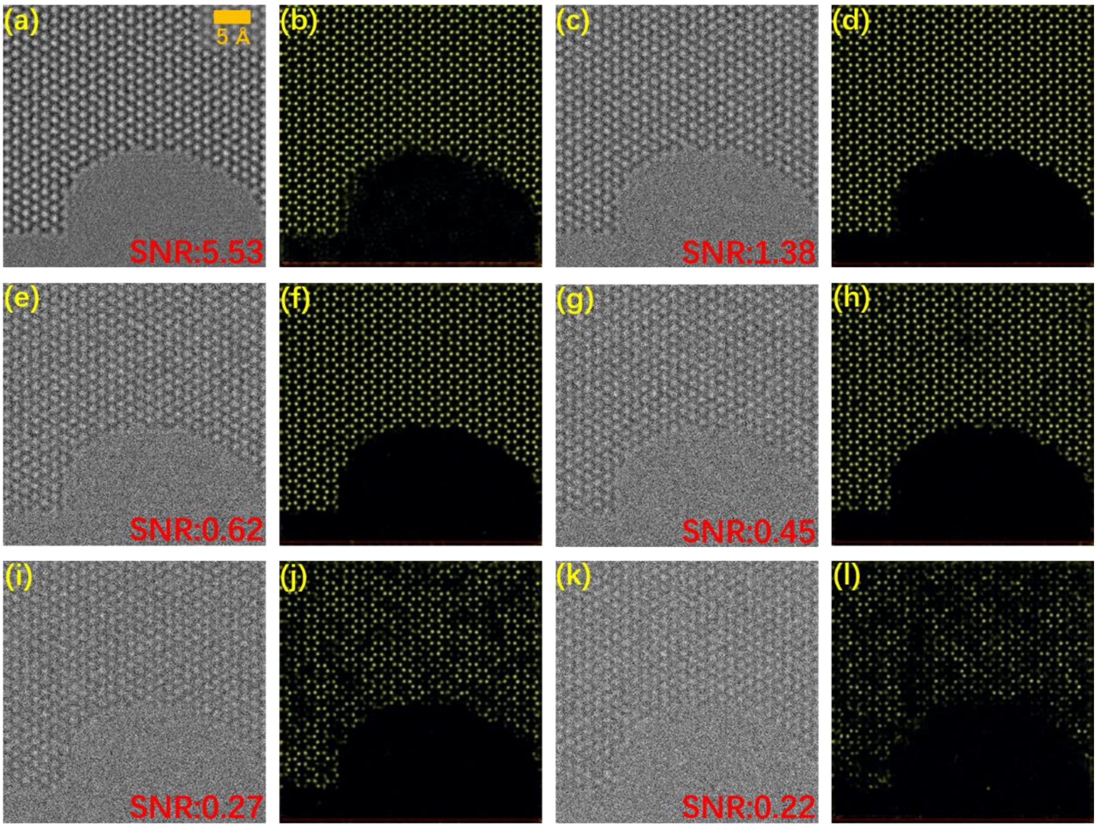

Supplementary Figure 4. Results of adding different levels of Poisson noise. (a, c, e, g, i, k) Simulated images and (b, d, f, h, j, l) their predictions with their SNRs varying. The SNRs are (a, b) 5.53, (c, d) 1.38, (e, f) 0.62, (g, h) 0.45, (i, j) 0.27, (k, l) 0.22.

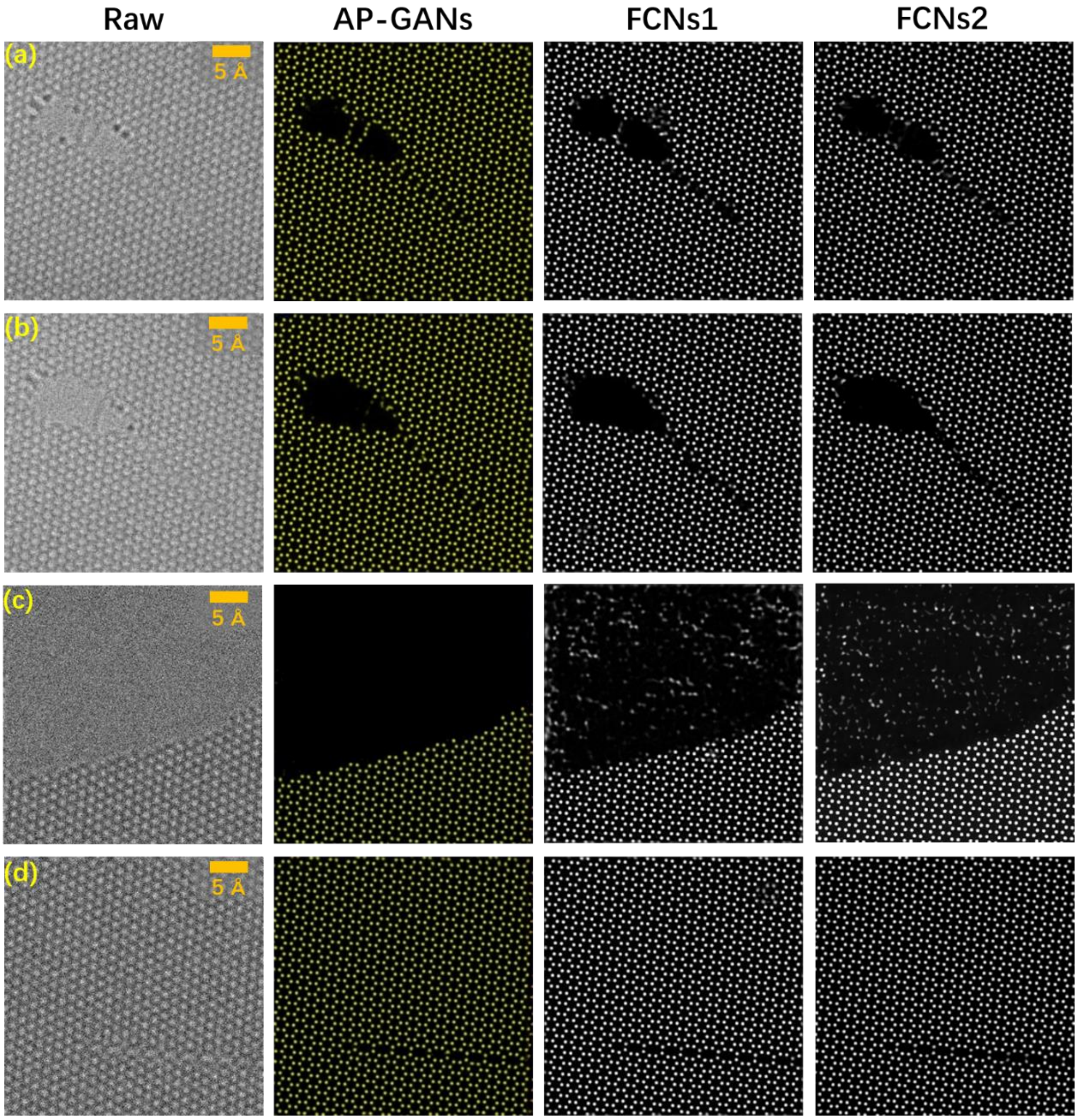

Supplementary Figure 5. Comparison of the prediction results of AP-GANs, FCNs1 and FCNs2 for the same experimental images in test set. (a-b) Experimental images and (c-d) simulated images in test set. The second, third and fourth rows were predicted via using the deep learning algorithms of AP-GANs, FCNs1 and FCNs2, respectively.

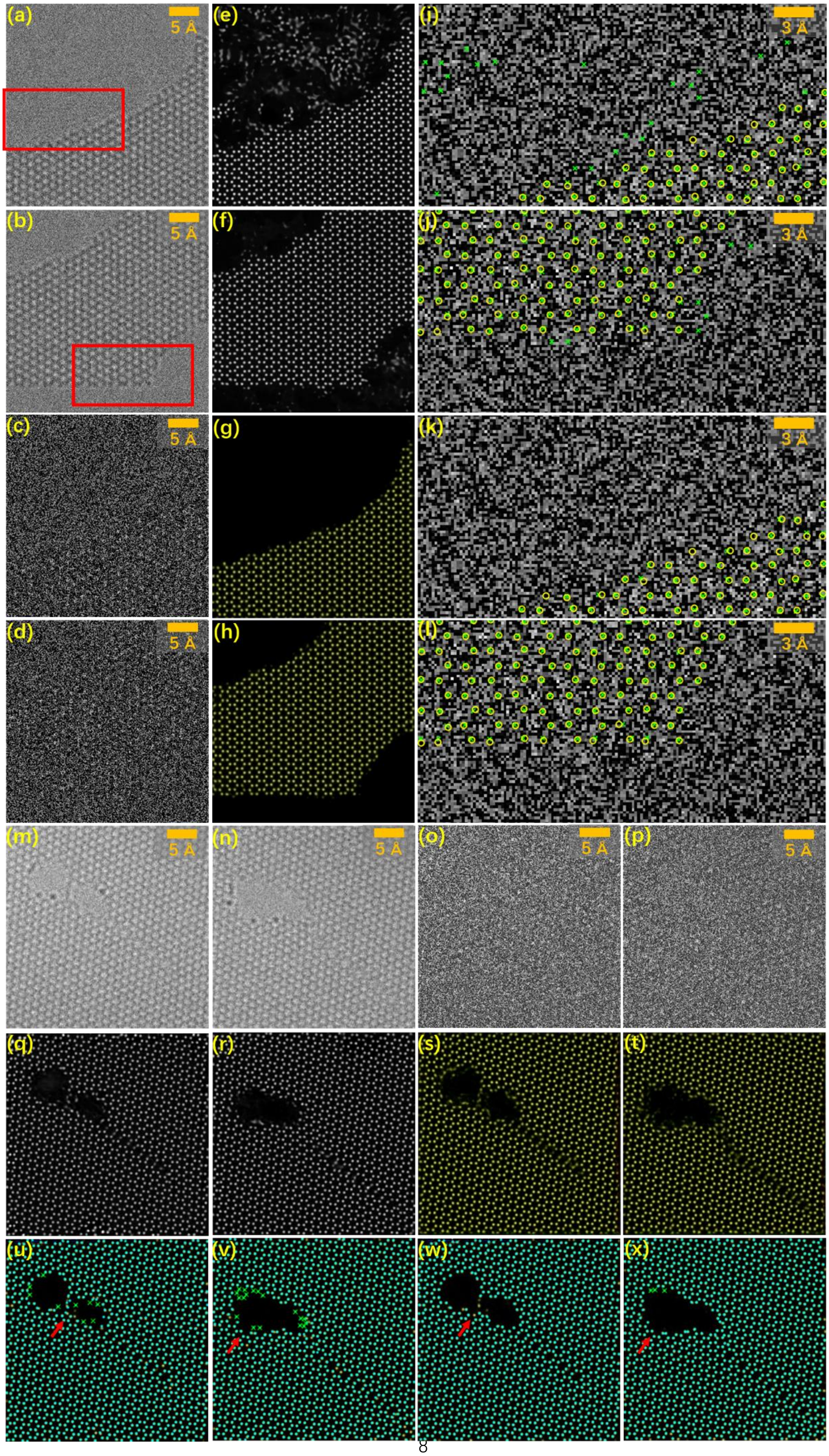
(a)
5 Å
(e)
(i)
3 Å
(b)
5 Å
(f)
(j)
3 Å
(c)
5 Å
(g)
(k)
3 Å
(d)
5 Å
(h)
(l)
3 Å
(m)
5 Å
(n)
5 Å
(o)
5 Å
(p)
5 Å
(q)
(r)
(s)
(t)
(u)
(v)
(w)
(x)

Supplementary Figure 6. Comparison of the predictions of AP-GANs and FCNs2 for the same HRTEM images in the test set. (a, b) Normal-dose simulated images, and (c, d) low-dose simulated images originated from (a, b), respectively. (e-h) Atom probability maps are predicted via using (e, f) FCNs2 and (g, h) AP-GANs, respectively. (i-l) Regions extracted from (a, b), with yellow circles quantified from (i, j) FCNs2 and (k, l) AP-GANs, respectively. And green crosses are the ground trues. (m, n) Normal-dose experimental images, and (o, p) low-dose experimental images originated from (m, n), respectively. (q-t) Atom probability maps are predicted via using (q, r) FCNs2 and (s, t) AP-GANs, respectively. (u-x) The comparison of atomic positions predicted from normal-dose images (m, n) and low-dose images (o, p), on which the darker yellow dots are estimated from normal-dose images via using AP-GANs and green dots are predicted from (q-t) respectively via our ARP and green crosses highlight clear artefacts.

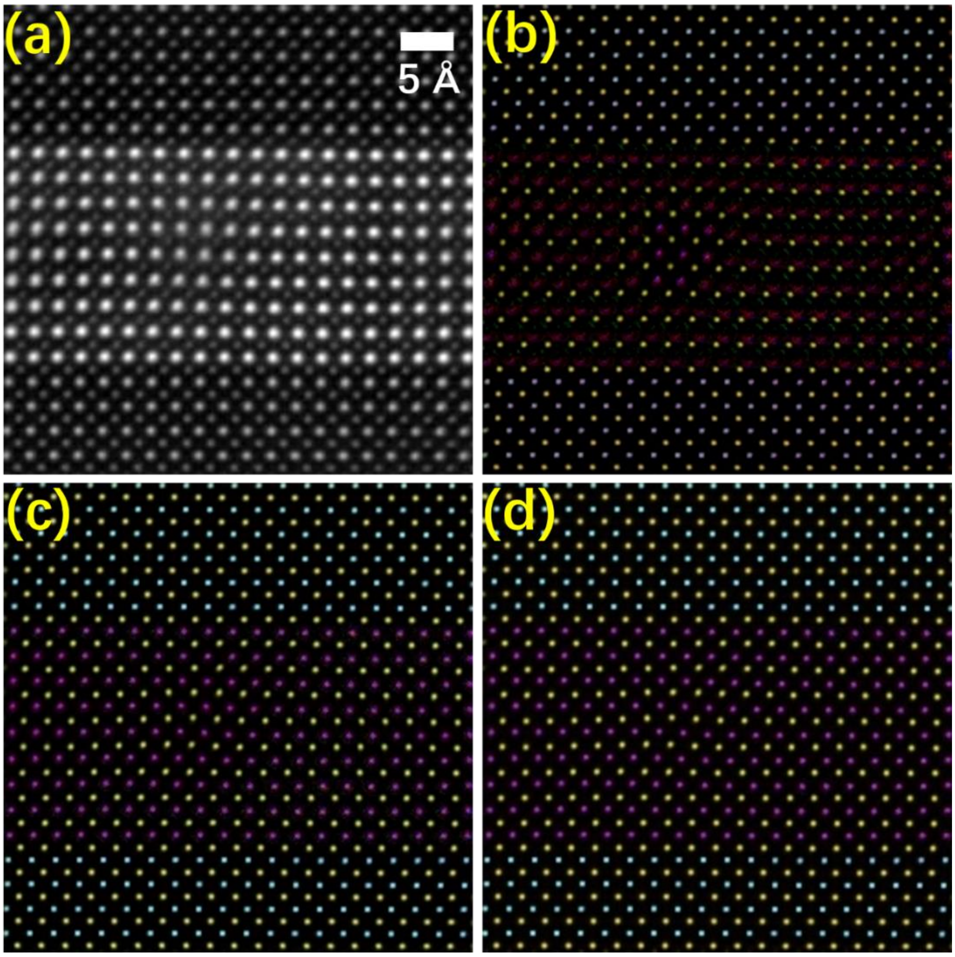

Supplementary Figure 7. (a) Normal-dose experimental image, and (b-d) elemental probability maps are predicted via using AP-GANs trained by (b) 10, (c) 20 and (d) 30 input-output pairs in training set.

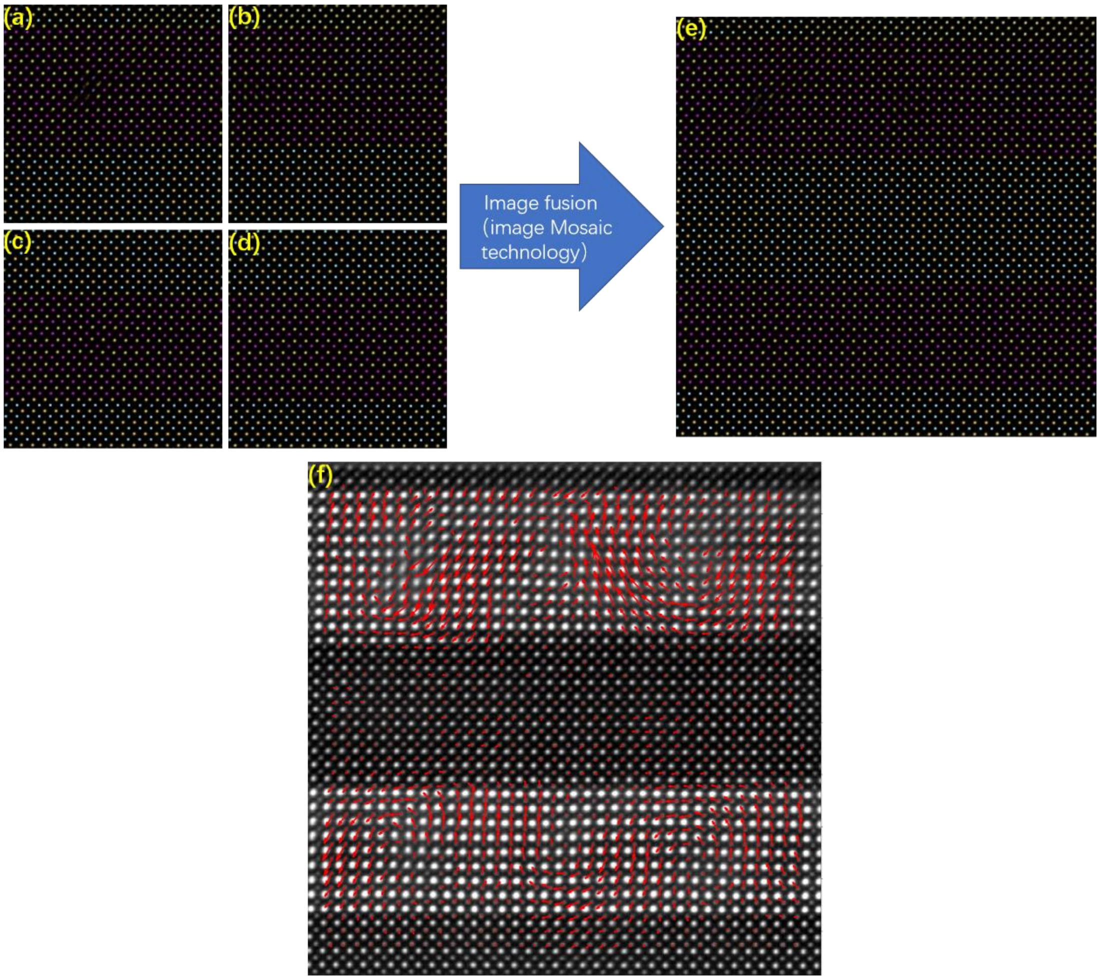

Supplementary Figure 8. (a-d) Four probability maps are stitched into (e) a big one. (f) Atomic displacement vector of Ti atoms quantified from (e).

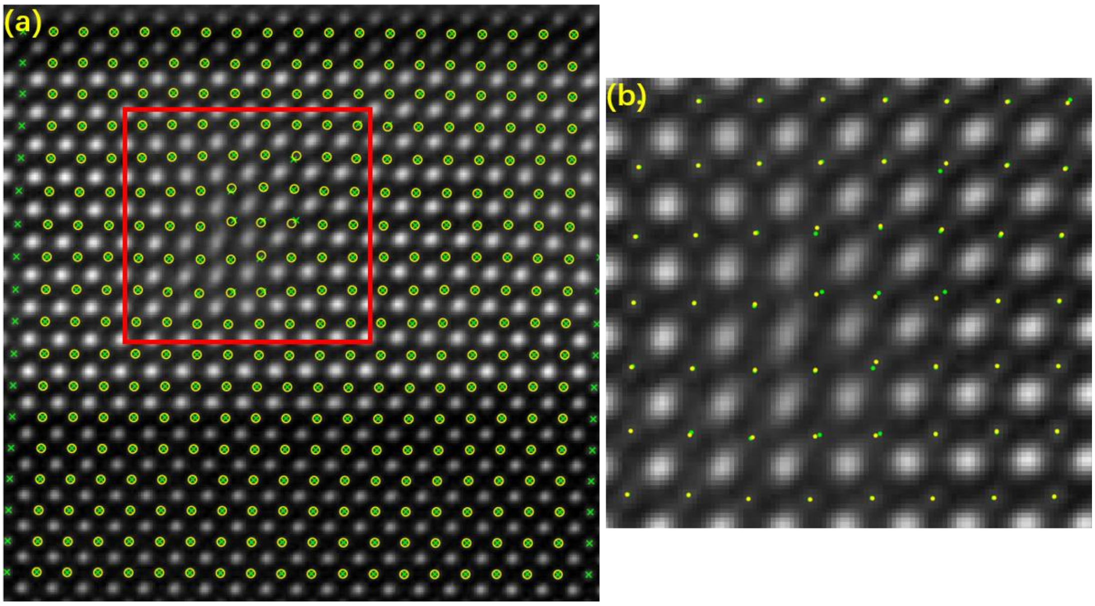

Supplementary Figure 9. (a) Raw images with only Ti atom positions marked. Yellow circles are positions of atoms quantified from the predicted probability map. And green crosses are positions labelled directly by CalAtom. (b) A region is extracted from the red box in (a) and dotted by dots of the same colors. The average intensity at yellow positions is $1.2694\times10^{5}\pm1.1292\times10^{4}$ (measured from the raw image), and that at green positions $1.2667\times10^{5}\pm1.1970\times10^{4}$, which means that the predicted positions is better than those directly measured from raw image (the accuracy is affected by the disturbance from the intensities of neighboring Pb atoms).

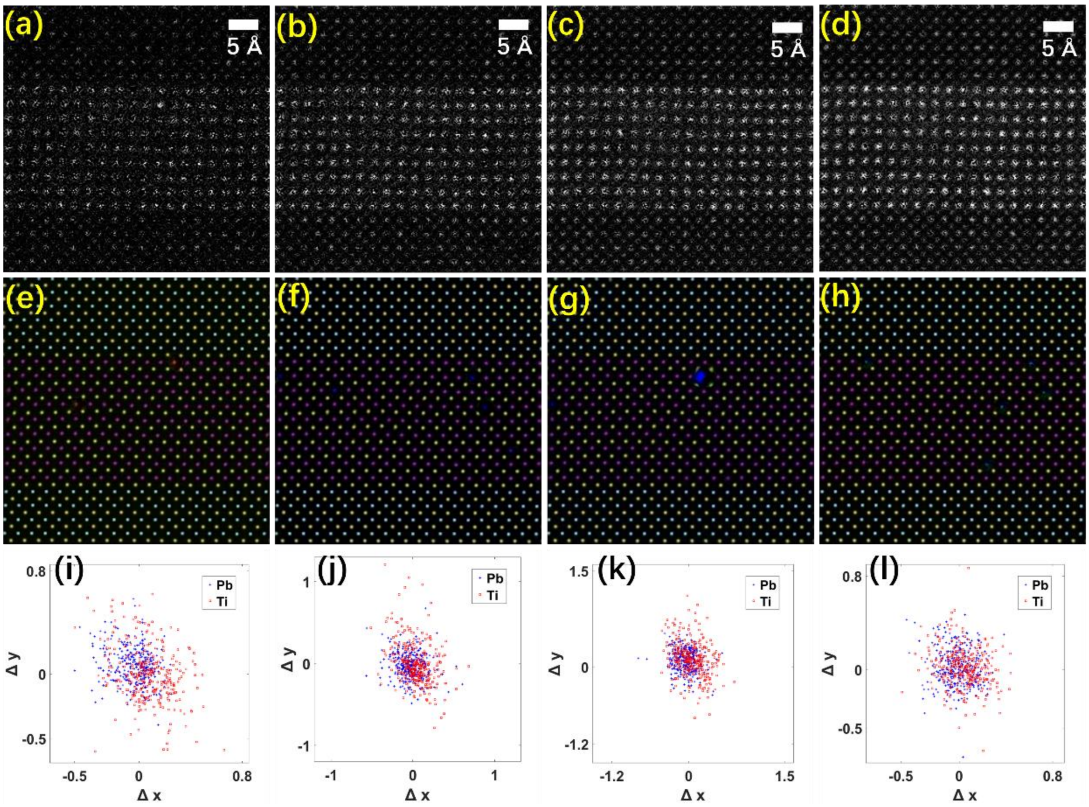

Supplementary Figure 10. (a-d) Low-dose images imitated from Fig. 7(a) in Supplementary Information and image intensities reserved with randomly (a) 30%, (b) 50%, (c) 70%, (d) 90% pixels, respectively. (e-h) Elemental probability maps predicted from (a-d) respectively. (i-l) Absolute displacements of all positions with units of pixels via comparing the elemental probability maps (e-h) with Fig. 7(d) in Supplementary Information. And red and blue dots represent the displacements of Ti and Pb/Sr sites, respectively.
